# Lipidomic analysis identifies age-disease-related changes and potential new biomarkers in brain-derived extracellular vesicles from metachromatic leukodystrophy mice

**DOI:** 10.1186/s12944-022-01644-8

**Published:** 2022-03-27

**Authors:** Melissa R. Pergande, Christina Kang, Diann George, Pearl A. Sutter, Stephen J. Crocker, Stephanie M. Cologna, Maria I. Givogri

**Affiliations:** 1 Department of Chemistry, University of Illinois Chicago, Chicago, IL 60607 USA; 2 Department of Anatomy and Cell Biology, College of Medicine, University of Illinois Chicago, 808 S. Wood St. M/C 512, Chicago, IL 60612 USA; 3 Department of Neuroscience, University of Connecticut School of Medicine, 263 Farmington Ave, Farmington, CT 06030 USA; 4 Laboratory for Integrative Neurosciences, University of Illinois Chicago, Chicago, IL 60607 USA

**Keywords:** Lipidomic, Extracellular vesicles, Mass spectrometry, Metachromatic leukodystrophy

## Abstract

**Background:**

Recent findings show that extracellular vesicle constituents can exert short- and long-range biological effects on neighboring cells in the brain, opening an exciting avenue for investigation in the field of neurodegenerative diseases. Although it is well documented that extracellular vesicles contain many lipids and are enriched in sphingomyelin, cholesterol, phosphatidylserines and phosphatidylinositols, no reports have addressed the lipidomic profile of brain derived EVs in the context of Metachromatic Leukodystrophy, a lysosomal storage disease with established metabolic alterations in sulfatides.

**Methods:**

In this study, we isolated and characterized the lipid content of brain-derived EVs using the arylsulfatase A knockout mouse as a model of the human condition.

**Results:**

Our results suggest that biogenesis of brain-derived EVs is a tightly regulated process in terms of size and protein concentration during postnatal life. Our lipidomic analysis demonstrated that sulfatides and their precursors (ceramides) as well as other lipids including fatty acids are altered in an age-dependent manner in EVs isolated from the brain of the knockout mouse.

**Conclusions:**

In addition to the possible involvement of EVs in the pathology of Metachromatic Leukodystrophy, our study underlines that measuring lipid signatures in EVs may be useful as biomarkers of disease, with potential application to other genetic lipidoses.

**Supplementary Information:**

The online version contains supplementary material available at 10.1186/s12944-022-01644-8.

## Background

There is an increasing interest to determine the role of extracellular vesicles (EVs) and their cargo in the pathogenesis of neurological conditions [1–4]. For example, pathogenic proteins in neurodegenerative diseases such as Alzheimer, Parkinson, and Lou Gehrig as well as some tauopathies are shuttled by EVs, with the possibility of inducing pathogenic effects on neighboring cells [5–11]. Because EVs are membranous vesicles, many lipids including sphingomyelin, cholesterol, phosphatidylserines, and phosphatidylinositols are also found in these vesicles [12, 13]. However, whether pathogenic lipids in genetic lipidoses contribute to disease remains unknown [14].

Sulfated glycolipids include, among others, sulfated galactocerebrosides (SM4s, sulfatides), sulfated lactosylceramides, and sulfated glucosylceramides. Sulfatides (3-O-sulfogalactosylceramides as referred in this study) are the main sulfated glycolipids accumulating in Metachromatic Leukodystrophy (MLD), a genetic, lysosomal storage disease (LSD) caused by deficiency of aryl-sulfatase A (ARSA). Sulfatides are known mediators of various biological processes, including regulating oligodendrocyte differentiation and myelination [15]. Sulfatides can be secreted within EVs from oligodendrocyte cultures [16] and are also found in plasma-derived EVs [17], among other biological fluids. Recently, we reported a significant increase of sulfatide lipids encompassing a 16-carbon fatty acyl chain in plasma-derived EVs isolated from Multiple Sclerosis patients, suggesting that the length of the acyl side chains may relate to specific disease conditions [17].

To further investigate the possibility that sulfatides are mobilized and contribute to pathogenesis in neighboring cells [14, 18], here we focused on the MLD mouse. The MLD mouse is a genetic knockout (*ARSA*^*−/−*^*)* which lacks expression of ARSA and progressively accumulates sulfatide lipids, primarily in white matter. MLD mice resemble some of the adult forms of the human disease with mild demyelination, motor deficiency, and only a slightly shortened lifespan [19, 20]. Despite earlier indications that sulfatides accumulate late (> 6 month old) in life of the MLD mouse, work from our laboratory and others showed that sulfatides are significantly accumulated already by the third month in some structures of the nervous system such as the cerebellar white matter [21]. Furthermore, recent studies using tandem mass spectrometry demonstrated that C16:0 and C18:0 sulfatides accumulate already during embryonic stages of MLD mice, suggesting a role in dysregulating the function of the Platelet Derived Growth Factor alpha (PDGFrα) mediated pathway [22].

In this study, we isolated brain-derived EVs from MLD mice (*ARSA*^*−/−*^) and WT control mice (*ARSA*^*+/+*^) and determined the concentration, particle size, protein content, and lipid content at multiple time points during disease progression. We found consistent similarities in EV size and protein concentration among all the time points evaluated between MLD and control mice. Using mass spectrometry, we performed relative quantification of multiple lipid classes extracted from EVs, including sulfatides (SHexCer), ceramides (Cer), fatty acids (FA), phosphatidylcholines (PC), phosphatidylglycerols (PG), phosphatidylethanolamines (PE), and triacylglycerols (TG). Our findings suggest that the lipid content of EVs may be involved in the brain pathology in MLD and present new lipid candidate biomarkers of the disease.

## Methods

### Reagents and chemicals

SPLASH Lipidomix was purchased from Avanti Polar Lipids (Alabaster, AL, USA). All other reagents and chemicals were acquired from Sigma-Aldrich (St. Louis, MO, USA) and used as received unless otherwise noted.

### Animal model and tissue collection

MLD mice were obtained from Dr. Ernesto R. Bongarzone (University of Illinois Chicago), originally generated by Dr. Volkmar Gieselmann (Bonn, Germany) [19]. Genotyping was performed by PCR from tail DNA as previously described [23]. Animals were maintained in standard housing conditions, and all experiments were performed according to institutional animal care committee- approved protocols (Protocol #16–088. Animal Welfare Assurance A3460.01). For both genotypes, *ARSA*^*+/+*^ (control mice) and *ARSA*^*−/−*^ *(*MLD mice*)*, three time points across the lifespan of the mice were selected: 30 days postnatal (P30), 3 months (3 m) and 6 months (6 m) of age. Although no significant difference in the amount of EVs between sexes has been observed, animals from both sexes were represented in each cohort. Animals were euthanized at P30, 3 m and 6 m of age (*N* = 3–5 for each genotype and age) and tissues were immediately harvested, flash frozen on dry ice and stored at -80 °C until use.

### Enrichment and characterization of EVs from brains tissue

Brain-derived EVs were collected from MLD and control mice employing a modified protocol developed by Perez-Gonzalez and colleagues [24]. Here, hemi sagittal brains were used where the tissue was first homogenized by blade dissociation in a papain solution (20 U/mL) for 1 min followed by incubation for 15 min in a water bath at 37 °C. The homogenate was then incubated on ice and protein and phosphatase inhibitors were added, after which homogenates were sequentially filtered through a 100 μm mesh filter followed by a 40 μm mesh filter. The filtrate was then subjected to a series of low (300 g for 10 min, followed by 2000 g for 10 min) and high-speed centrifugations (10,000 g for 30 min, twice, then 100,000 g for 90 min). Next, some EVs preparations were resuspended in phosphate buffered saline solution (PBS) and loaded into a sucrose gradient of 0.25 M up to 2 M in 0.35 M increments. The sucrose gradient was subjected to ultracentrifugation at 200,000 g for 16 h, after which 6 fractions were collected (labeled A-F). Lastly, individual fractions were centrifuged at 100,000 g to concentrate the EVs. The final pellet was resuspended in 90 μL of ice-cold PBS and analyzed by Nanosight, protein content (BCA kit) and Western Blot.

### Nanosight analysis of EVs

EVs were characterized using Nanoparticle Tracking Analysis (NTA). EV concentration and particle size was determined using a NanoSight NS300. The EV preparations were diluted 500-fold with 0.1 μm filtered, sterile PBS to make a final volume of 1 mL and loaded in a syringe placed on a syringe pump which was attached to the instrument. Three movies were acquired for each sample using NS300 software (v3.2) using the following settings: temperature 22.4–22.6 °C, capture duration 30s/video, camara level 8 and detection threshold 7 [25].

### Transmission electron microscopy

Negative staining was performed using 15uL of each EV sample that was resuspended in sterile PBS. Droplets were absorbed onto activated copper grids with carbon coating (Electron Microscopy Sciences, Hatfield, PA, USA) for 15 min, as previously described [26]. Grids were washed three times (in deionized water) and then stained with 1% uranyl acetate (Electron Microscopy Sciences) for 1 min. Excess uranyl acetate was removed by gently blotting each grid before drying overnight. Samples were then imaged using the Hitachi H-7650 transmission electron microscope.

### Western blot analysis

Gel electrophoresis was performed using precast gradient 4–12% polyacrylamide gels using the XCell Sure-lock vertical electrophoresis system (Thermo Fisher Scientific, Waltham, MA). Proteins were wet electrotransferred to PVDF membranes. The membranes were blocked with a 5% non-fat milk solution (prepared in TBS-Tween-20) for 1 h at room temperature and incubated at 4 °C overnight with primary antibody. Peroxidase-labeled secondary antibodies were used to detect antibody-reactive products by ECL chemiluminescent substrate (Thermo Fisher Scientific, Waltham, MA). Western Blots for flotillin 1 (FLOT1), Ras-related protein Rab-5B (RAB5B), programmed cell death 6-interacting protein (ALIX), disintegrin and metalloproteinase domain-containing protein 10 (ADAM10), early endosome antigen 1 (EEA1), calnexin (CALX) and synaptotagmin 1 (SYT1) were assessed in enriched EV preparations to evaluate possible contamination with other cellular organelles. Also, cell specific markers including: myelin basic protein (MBP,oligodendrocyte marker), glial fibrillary acidic protein (GFAP, astrocyte marker), allograft inflammatory factor 1 (IBA1, microglia marker) and beta-tubulin III (TUBB3, neuronal marker) were evaluated. Additionally, major antigen presenting class II levels (MHCII, antigen presenting cells) were evaluated as well as actin and sodium/potassium-transporting ATPase subunit beta-1 (ATP1B1) for housekeeping proteins. Information for all antibodies, including dilutions and vendors, can be found in Additional file 1. Densitometry analysis was done utilizing ImageJ software (Additional file 2).

### Extraction and LC-MS analysis of EVs lipid extracts

Isolated EVs were re-suspended in PBS buffer, probe sonicated and the protein concentration determined using a bicinchoninic acid assay (BCA). Lipid extracts from the PBS homogenates (100 μg protein equivalent) were prepared for each of the EV samples via liquid-liquid extraction using the Folch method [27]. Prior to extraction, 10 μL of SPLASH Lipidomix was spiked in as an internal standard. The resulting lipid extracts were dried in vacuo and resuspended in 100 μL of methanol: chloroform (9:1, v/v) prior to analysis. Given that chloroform is not generally preferred for the use with LC tubing such as Peak, several resuspension experiments with different organic solvents including methanol, chloroform and even toluene were performed. We found that the combination used in this study is able to yield sufficient solubility of extracted lipid species to achieve identification and subsequent relative quantification of numerous lipid species. Mass spectrometry analysis of the crude lipid extracts was performed using an Agilent 6545 Q-TOF liquid chromatography mass spectrometry (LC-MS) system controlled by the Agilent Mass Hunter acquisition software as previously described [28] for data dependent analysis. Source parameters were as follows: gas temperature 200 °C, drying gas flow 11 L/min, nebulizer pressure 35 psi, sheath gas temperature 350 °C, sheath gas flow 12 L/min, capillary voltage 3000 V, nozzle voltage 1000 V and fragmentor voltage 175 V. Data was collected for relative quantification using a scan speed of 3 MS spectra per second. A pooled sample was prepared by combining 5 μL of each of the EV lipid extracts and an iterative MS/MS workflow was performed in the Mass Hunter acquisition software across 8 injections of the pooled samples with a scan speed of 3 MS and 3 MS/MS spectra per second of the top 5 precursors. All raw mass spectrometry data is publicly available at ftp://massive.ucsd.edu/MSV000086647/.

### LC-MS data analysis

Lipids assignments were made based on fragmentation matching (MS/MS) to the in-silico LipidBlast library using the Lipid Annotator software (Agilent Technologies Inc., Santa Clara, CA, USA) using the following settings for identifications: lipid species for positive ([M + H]^+^, [M + Na]^+^, [M + NH_4_]^+^, [M + H-H_2_O]^+,^ [M + Na-H_2_O]^+^, [M + NH_4_-H_2_O]^+^) and negative ([M-H]^−^, [M + CH_3_COO]^−^, [M-H-H_2_O]^−^, [M + CH_3_COO-H_2_O]^−^) ions, Q-Score > 60, and mass deviation < 5 ppm. Raw LC-MS data files were processed using the Profinder software (vB.08.00, Agilent Technologies Inc., Santa Clara, CA, USA). Molecular features were extracted for peaks > 10,000 counts using an isotope model of common organic molecules (no halogens). The resulting compound list was further filtered for even chain lipids and compounds having two or more isotopes present. Additionally, retention time for each compound was aligned to ±0.1 min using a mass accuracy window of < 5.0 ppm and extracted ion peaks integrated using the Agilent integrator in the Profinder software. Each of the integrated peaks was manually reviewed for retention time and fragmentation matching. Each processed data file was then exported as a.cef file and imported into the Mass Profiler Professional software (v15.1, Agilent Technologies Inc., Santa Clara, CA, USA) where each data set was analyzed separately in positive and negative ion modes. Additionally, a lipid class-based normalization workflow was performed in the Mass Profiler Professional software to account for any variation in extraction efficiency across samples. In both cases, all compound intensities were baselined to median intensity. Compounds that were not present in all biological replicates of either genotype were further filtered. Next, a list of altered compounds for each ion mode was generated based on individual lipid species intensities where an unpaired t-test (*p* < 0.05) was used to determine significant alterations at each timepoint of disease between genotypes (i.e., P30 control mice vs P30 MLD mice*,* 3 m control mice vs 3 m MLD mice and 6 m control mice vs 6 m MLD mice) for both positive and negative ionization modes. All reported lipid identifications follow the nomenclature of LIPID MAPS Lipid Classification System [29, 30] (https://www.lipidmaps.org/).

### Statistical analysis

The analysis of Nanosight data was performed using the Graphpad Prism 9 software macOS (9.1.2). One-way Anova with Tukey multiple comparison test was used to analyze statistics of EV between genotypes for the three-time points evaluated. Additionally, an unpaired T-test was used to evaluate a single time point difference among genotypes. Significantly altered lipid species was determined via an unpaired t-test of normalized species intensities in the Mass Profiler Professional software (v15.1, Agilent Technologies Inc., Santa Clara, CA, USA).

## Results

**Fig. 1:**
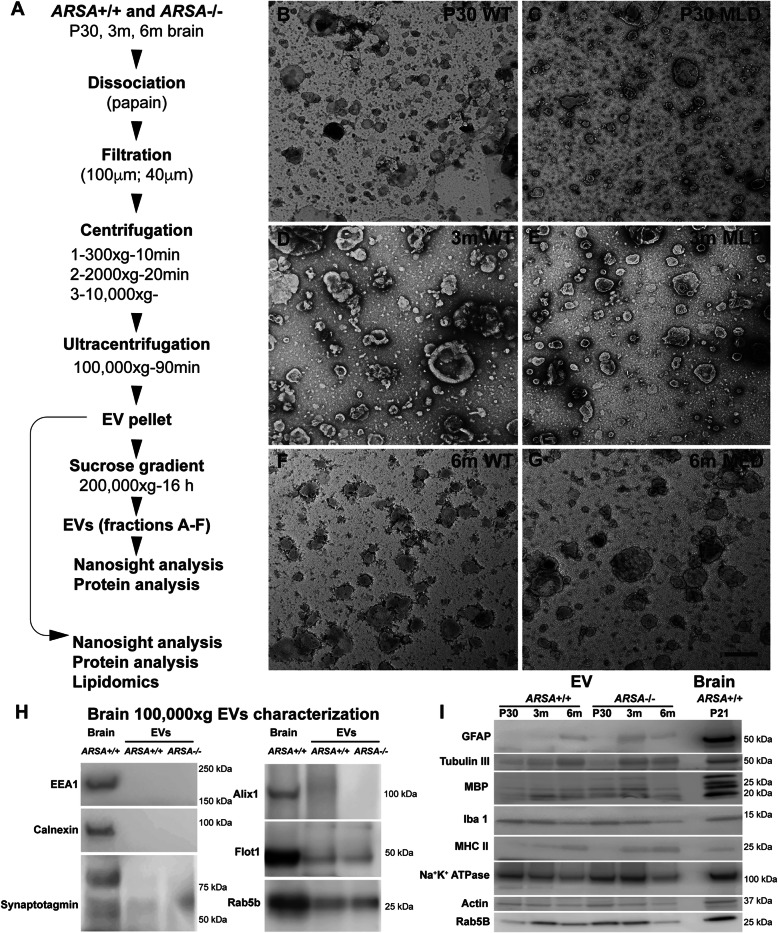
Enrichment and characterization of brain derived extracellular vesicles from MLD mice. **A**) Schematic of the workflow used to enrich extracellular vesicles (EVs). Total EVs were enriched from brain tissue from MLD and control mice at 30 postnatal days (P30), 3 months (3 m) and 6 months (6 m) of age. **B**-**G**) Transmission electron microscopy of isolated EVs. Showing representative imagines obtained from WT (*ARSA*^*+/+*^) vs MLD (*ARSA*^*−/−*^) EVs from postnatal day 30 (P30), 3 and 6 months (3 m and 6 m). **H**) EV samples were subjected to electrophoresis and Western blot analysis for common cellular vesicle markers. Representative images obtained from enriched EVs samples isolated from brains of 6 m revealed little detection of early endosome antigen 1 (EEA1), endoplasmic reticulum calnexin (CALX) and synaptic synaptotagmin 1 protein (SYT1) (*n* = 1 per genotype and time point). EVs markers flotillin 1 (FLOT1) and ras-related protein Rab-5B (RAB5B) were detected and programmed cell death 6-interacting protein (ALIX) was absent. **I**) Qualitative analysis by western blot analysis of cell type specific markers showed reactivity for glial fibrillary acidic protein (GFAP, astrocytes), beta-tubulin III (TUBB3, neurons), myelin basic protein (MBP, oligodendrocytes), allograft inflammatory factor 1 (IBA1, microglia) and MHCII. Also, reactivity for Actin and sodium/potassium-transporting ATPase subunit beta-1 (Na + K+ ATPase) were detected (*n* = 1 per genotype and time point)

Brain-derived EVs were isolated from 190 to 250 mg of brain material from MLD and control mice at each time point: P30, 3 m and 6 m of age. EVs were prepared both as total EVs or subsequently fractionated by size-based subpopulations via sucrose gradient centrifugation (Fig. 1A) for characterization. First, the EV preparations were examined by transmission electron microscopy (TEM) to verify the structure in our sample preparations (Fig. 1B-G). As seen in Fig. 1 the often appearing cup shape of EVs was apparent and of different sizes, as was expected. EVs preparations from 3 m brains seemed to be larger in size than the ones obtained at P30 and 6 m (Fig. 1D,E). Western blot analysis of the total EV preparation was performed to evaluate the enrichment efficiency (Fig. 1H). EV markers including FLOT1 and RAB5B, known to be enriched in EVs [31, 32], were confirmed in our preparations (Fig. 1H). ALIX and tumor susceptibility gene 101 protein (TSG101), also often seen in some EV preparations [33], were absent (data not shown). Non-EV markers such as EEA1 (early endosomal) and CALX (endoplasmic reticulum) were not detected in either genotype preparations (Fig. 1H); however, a small reactivity for synaptotagmin (synaptic vesicles) in EVs from both genotypes was observed (Fig. 1H). To study whether the EVs originate from specific neural cell types, western blotting for neural cell-type markers was done for MBP (oligodendrocytes), TUBB3 (neurons), IBA1 (microglia), GFAP (astrocytes) and MHCII (antigen presenting cells) (Fig. 1I). Our analysis revealed the presence of MBP, IBA1 and tubulin III in all samples, with minor immunoreactivity for GFAP and MHCII in EVs (Fig. 1I, Additional file 2). These results suggest that total EVs represent heterogenous vesicle populations derived from most, if not all, neural cell types.

**Fig. 2:**
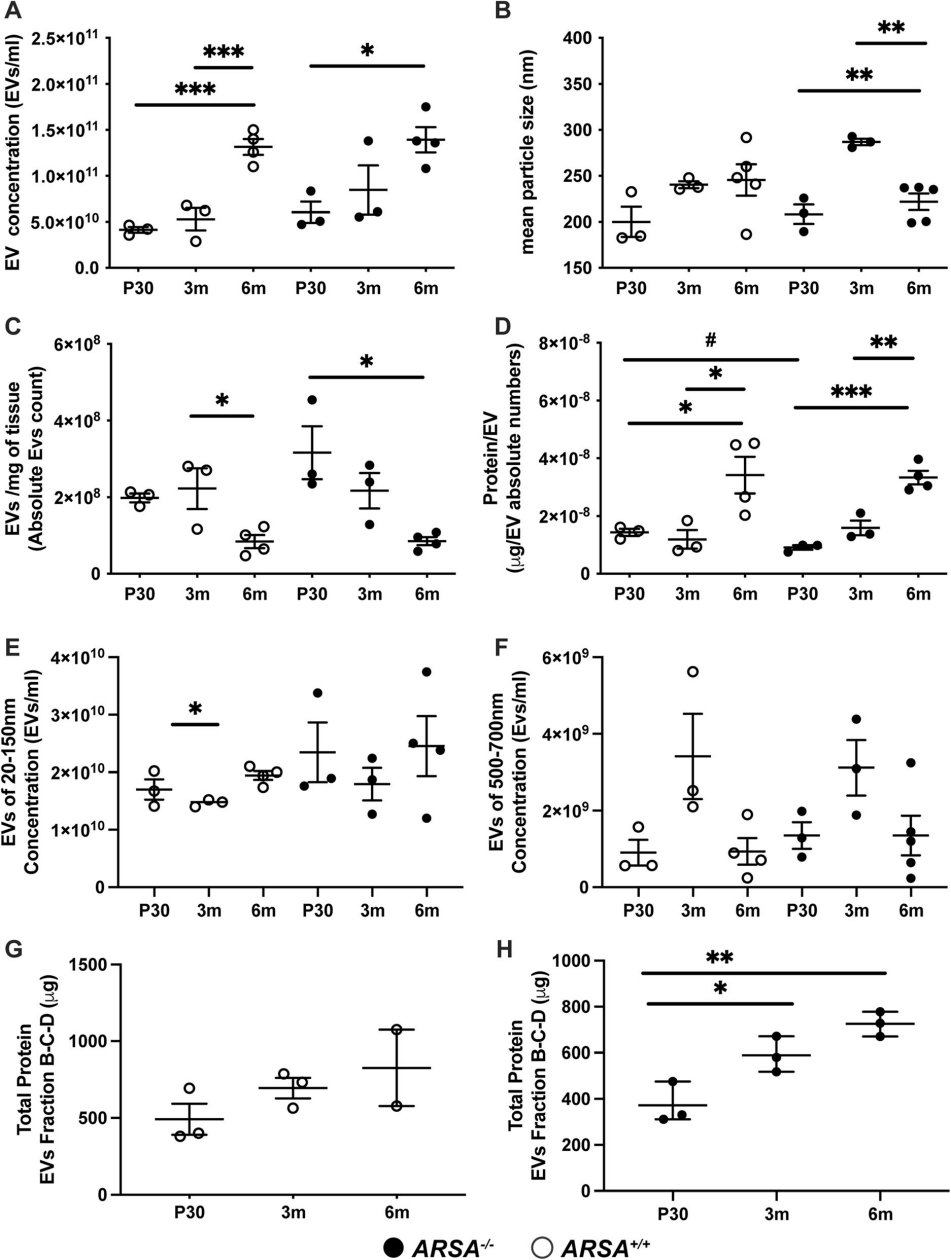
Characterization of isolated extracellular vesicles from MLD and control mice by Nanosight tracking analysis. Nanosight tracking analysis (NTA) of extracellular vesicles (EVs) pelleted at 100,000 g from mice at 30 postnatal days (P30), 3 months (3 m) and 6 months (6 m) of age. Plots showing **A**) EV concentration, **B**) EV mean particle size, **C**) number of EVs per milligram of brain tissue, **D**) total protein, **E**) concentration of EVs (measured 20-150 nm) and F) concentration of EVs (measured 500-700 nm). Protein content was measured from EVs isolated after a sucrose gradient and pooling vesicles from fractions **B**, **C** and **D** (Fig. 1) in **G**) WT (*ARSA*^*+/+*^) and **H**) MLD (*ARSA*^*−/−*^) EVs. White circles, WT EVs and black circles represent MLD EVs. Error bars represent mean ± SEM, *n* = 3–5 biological replicates and 3 technical replicates per genotype and time point. Significance was determined by a one-way Anova and Turkey’s multiple comparison (*p* < 0.05 = *; *p* < 0.001**; *p* < 0.0001 = ***) and Unpaired T-test (# *p* = 0.029)

Next, we considered the size-distribution of EVs enriched in our preparation. The NTA data revealed novel information regarding the concentration (EVs/mL of PBS) and size of EVs in the brains of MLD and control mice (Additional file 3). Remarkably, the concentration of EVs appears to be tightly controlled in both genotypes at each timepoint (Fig. 2A), where EV concentration seems to increase in parallel with age. Within a specific age group of control mice, EV concentration was found to be significant between P30 and 6 m animals (one-way ANOVA; *p* = 0.004) and between 3 m and 6 m animals (one-way ANOVA; *p* = 0.001). Evaluation of MLD mice revealed differences between P30 and 6 m animals (one-way ANOVA; *p* = 0.036). Moreover, NTA analysis showed the presence of size distributions of EVs, with a mean particle size of 200 nm for control mice at P30 and 250 nm at 3 m and 6 m. The mean particle size for MLD mice was 200 nm at P30, 300 nm at 3 m and 220 nm at 6 m (Fig. 2B and Additional file 4). The difference in EV size for MLD mice was significant at 3 m with respect to other time points of disease (one-way ANOVA; *p* < 0.002).

When the concentration of EVs was corrected for the applied dilution factor, the volume of suspension of isolated EVs and the weight of brain tissue, a striking difference was observed (Fig. 2C). Here, a significant decrease in the concentration of EVs between 3 m to 6 m control mice (one-way ANOVA *p* = 0.031) and between P30 and 6 m from MLD mice was measured (one-way ANOVA, *p* = 0.015). Interestingly, when protein concentration was measured, significant variations were found between times points among the different genotypes (Fig. 2D). EVs from both genotypes progressively and significantly increased in protein content with increasing age when comparing EVs from control mice between P30 and 6 m (one-way ANOVA; *p* = 0.05) and between 3 m and 6 m (one-way ANOVA; p = 0.03). Moreover, comparing MLD derived EVs between P30 and 6 m (one-way ANOVA; *p* = 0.0002) and 3 m and 6 m (one-way ANOVA; *p* = 0.0018), significant differences were observed. Protein content was lower in EVs from MLD mice and significantly different from EVs from control mice only at P30 (unpaired t-test; *p* = 0.022). The concentration for small EVs (20–150 nm) and medium sized EVs (500-700 nm) for each genotype was also evaluated (Fig. 2E and F). Small EVs enriched from control mice were significantly increased in their concentration from 3 m to 6 m age (one-way ANOVA; *p* = 0.034); no differences were observed in EVs isolated from MLD mice*.* No significant difference was observed for medium sized EVs in either genotype.

To further characterize the subpopulations of vesicles enriched in the total EV preparation, samples were also subjected to a sucrose gradient centrifugation which resolved EVs into six fractions (A-F) as depicted in Fig. 1A. The so-called “exosome fraction” was previously shown to be enriched in fractions B, C and D when a 0.22 μm pre-filtration step was carried out [24]. In this study, sucrose fractions were subjected to NTA (Additional files 5 and 6) and protein concentration analysis (Fig. 2G and H). In our hands, we found that fractions A, E and F lack significant levels of protein. Instead, the protein concentration was found to be similar for fractions B, C, and D among the three time points for both genotypes. Interestingly, fraction C showed the maximum protein content for both genotypes. No significant difference in protein concentration was observed between genotypes for these sucrose fractions. On the other hand, a significance difference was measured in the sucrose fractions of EVs enriched from MLD mice between P30 and 3 m and 6 m (Fig. 2H; one-way ANOVA; *p* = 0.027 and *p* = 0.0029, respectively).

**Fig. 3:**
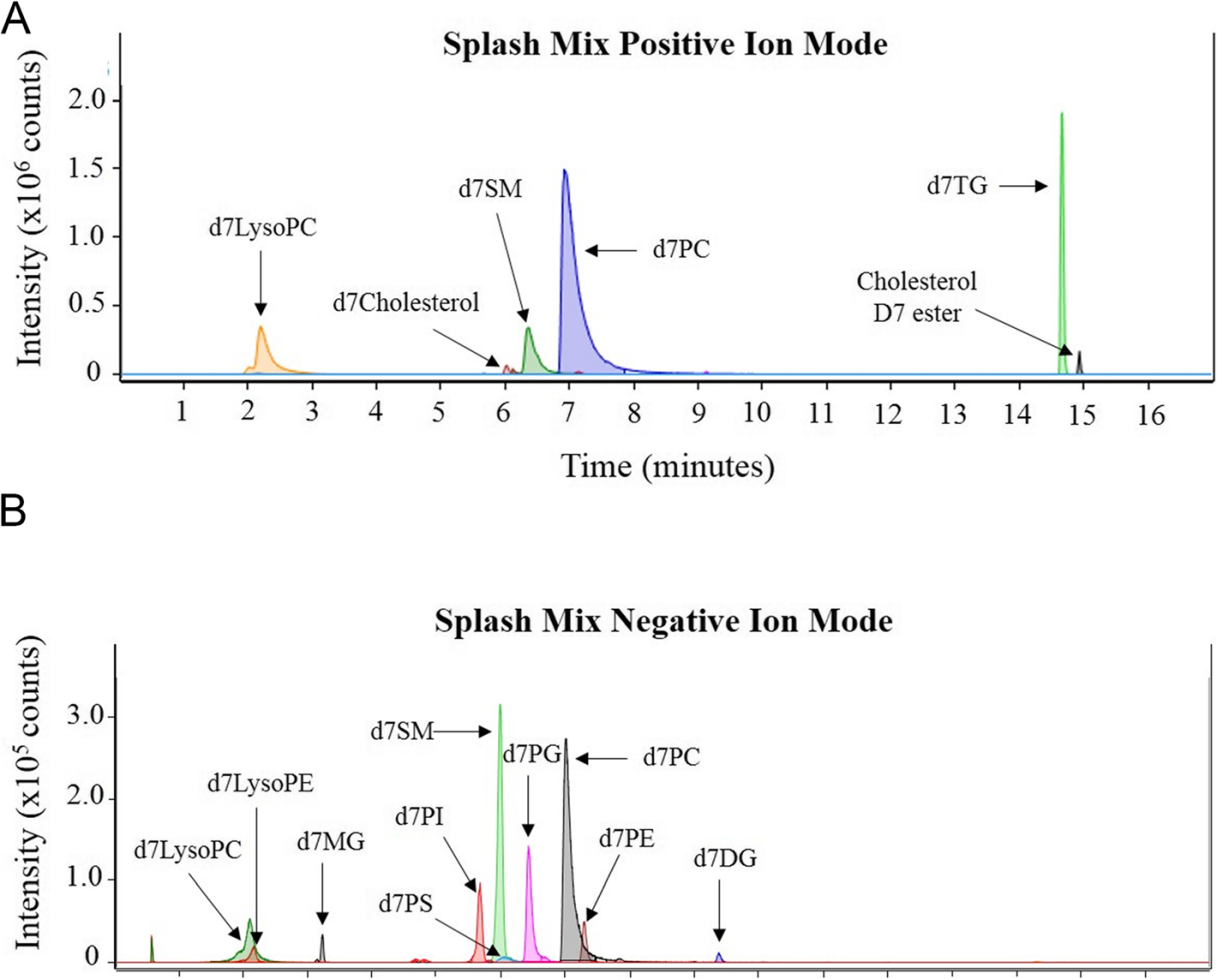
Analysis of internal standard SPLASH lipidomix in positive and negative ion mode. Extracted ion chromatograms displaying retention times of internal standards detected in positive (**A**) and negative (**B**) ionization modes

Following the initial EV characterization, an untargeted lipidomic analysis was carried out on EVs enriched from both genotypes. In this study, a total of 282 and 225 lipids were identified via MS/MS fragmentation in positive and negative ion modes, respectively (Additional files 7 and 8). In reversed-phase liquid chromatography, each lipid class tends to have a characteristic retention time window [28, 34, 35]. With this information in hand, the altered lipid species in each class were compared to a retention time standard, when possible, for retention time matching (Fig. 3). Heatmaps for measured intensities of all lipids identified by fragmentation matching can be reviewed in Additional file 9. Based on the measured intensities, the relative abundance of all lipid species was determined between genotypes. We found 14, 25, 120 lipid species to be altered in positive mode at the P30, 3 m and 6 m time points. Similarly, 10, 9 and 63 lipids were found to be significantly altered in negative ion mode at P30, 3 m and 6 m of age, respectively when comparing MLD to controls (Additional files 7, 8 and 10).

**Fig. 4:**
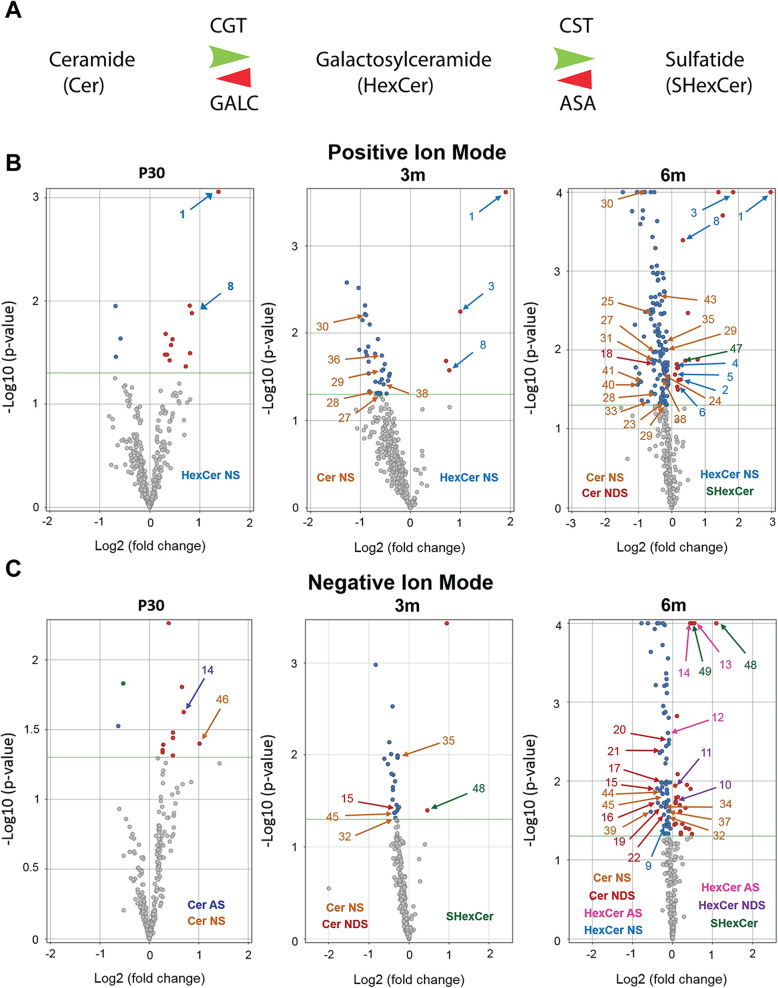
Ceramides, Hexocylceramides and sulfatides are altered in brain derived EVs enriched from *ARSA*^*−/−*^ mice. **A**) Graphical representation of enzymes involved in the synthesis of ceramides (Cer), Hexosylceramide (HexCer) and sulfatide (SHexCer). CGT: cerebroside galactosyl transferase; GALC: galactosylceramidase (also known as beta-galactocerebrosidase); CST: cerebroside sulfo transferase and ARSA: Arylsulfatase A. **B**-**C**) Volcano plots highlighting significantly (*n* = 3 per genotype and time point; *p* < 0.05) altered Cer, HexCer and SHexCer lipids observed in brain-derived EVs from MLD relative to control mice. Comparisons were done between lipidomic analysis of EVs from 30 postnatal days (P30), 3 months (3 m) and 6 months (6 m) of age in positive (B;top) and negative (C;bottom) ionization modes. Annotated are ceramide non-hydroxyfatty acid sphingone (Cer_NS, orange), ceramide alpha-hydroxy fatty acid-sphingosine (Cer_AS, dark blue), ceramide non-hydroxyfatty acid-dihydrosphingosine (Cer_NDS, red), hexosylceramide non- hydroxyfatty acid-phingosine (HexCer_NS,light blue),hexosylceramide alpha-hydroxy fatty acid-sphingosine (HexCer_AS, pink) and sulfatide (SHexCer, green). The key for number lipid species is presented in Table 1. *n* = 3 biological replicates per genotype and time point. Significance was determined via an unpaired t-test (*p* < 0.05, * *p* < 0.01, **; *p* < 0.001, ***)

Due to lack of ARSA expression in MLD mice, sulfatides have been reported to begin accumulating in early time points of the disease [22]. With this expectation, the relative abundances of sulfatides, specifically, SHexCer and their chemical precursors, ceramides (Cer) and hexosylceramides (HexCer) as shown in Fig. 4A were evaluated and found to be altered in MLD mice. With this, volcano plots were generated to visualize the relative abundance of these lipids in the EVs enriched from both genotypes at P30, 3 m and 6 m time points. A significant increase in sulfatides was observed as expected in MLD mice at 6 m of age (Fig. 4 B-C, detailed information on lipid ID identification numbers present at volcano plots are found in Table 1). At P30, a significant increase in HexCer, non-hydroxyfatty acid-sphingosine (HexCerNS), ceramide non-hydroxyfatty acid-sphingosine (CerNS) and ceramide alpha-hydroxy fatty acid-sphingosine (CerAS) was observed in EVs enriched from MLD mice relative to control (Fig. 4B and C; lipid identification found in Table 1). At 3 m, a significant decrease in CerNS and of ceramide non-hydroxyfatty acid-dihydrosphingosine (Cer_NDS) along with a significant increase in HexCe_rNS and SHexCer was observed in MLD EVs relative to control (Fig. 4B and C). Finally, at 6 m, a significant decrease in Cer_NS, Cer_NDS, hexosylceramide alpha-hydroxy fatty acid-sphingosine (HexCerAS), HexCerNS and a significant increase in HexCer_NS, HexCer_AS and HexCer_NDS was observed in MLD EVs relative to control (Fig. 4B and C, lipid identifications found in Table 1).

**Table 1:** Lipids identified from EVs by differential lipidomics in MLD versus control

ID	Compound	Fold Change of Normalized Intensities (***MLD*** vs. ***Control)***		
		P30	3 m	6 m
**1**	**HexCer_NS d18:1_16:0 (+)**	2.6***	3.8***	7.9***
**2**	**HexCer_NS d18:1_18:0 (+)**	**−1.2**	**− 1.1**	1.2 *
**3**	**HexCer_NS d18:1_18:1 (+)**	**1.5**	2.0 **	3.6***
**4**	**HexCer_NS d18:1_22:0 (+)**	**1.0**	**−1.3**	1.1 *
**5**	**HexCer_NS d18:1_22:1 RT:10.345 (+)**	**−1.1**	**− 1.3**	1.1 *
**6**	**HexCer_NS d18:1_22:1 RT:9.311 (+)**	**−1.0**	**− 1.2**	1.1 *
**7**	**HexCer_NS d18:1_24:1 (+)**	**−1.1**	**−1.4**	− 1.1 *
**8**	**HexCer_NS d18:2_18:0 (+)**	1.8 *	1.7 *	2.9***
**9**	**HexCer_NS d42:3 (−)**	**1.0**	**−1.5**	−1.2 *
**10**	**HexCer_NDS d40:1 (−)**	**1.1**	**−1.3**	1.1 *
**11**	**HexCer_NDS d40:2 (−)**	**1.0**	**−1.1**	1.1 *
**12**	**Cer_AS d36:1 (−)**	**1.2**	**−1.2**	−1.1 **
**13**	**Cer_AS d40:1 (−)**	**1.5**	**1.5**	2.1***
**14**	**Cer_AS d42:1 (−)**	1.6 *	**1.7**	1.8***
**15**	**Cer_NDS d16:0_26:2 (−)**	**−1.0**	−1.6 *	−1.4 *
**16**	**Cer_NDS d18:0_16:0 (−)**	**−1.1**	**−1.3**	− 1.6 *
**17**	**Cer_NDS d18:0_18:0 (−)**	**1.1**	**−1.5**	−1.5 *
**18**	**Cer_NDS d18:0_18:0 (+)**	**−1.1**	**−1.5**	− 1.5 *
**19**	**Cer_NDS d18:0_22:0 (−)**	**−1.1**	**− 1.3**	−1.3 *
**20**	**Cer_NDS d20:0_18:0 (−)**	**1.0**	**−1.3**	−1.1 **
**21**	**Cer_NDS d40:1 (−)**	**1.0**	**−1.5**	−1.4 **
**22**	**Cer_NDS d42:1 (−)**	**1.0**	**−1.5**	−1.3 *
**23**	**Cer_NS d18:1_14:0 (+)**	**−1.7**	**−1.1**	− 1.2 *
**24**	**Cer_NS d18:1_18:0 (+)**	**−1.1**	**−1.3**	− 1.2 *
**25**	**Cer_NS d18:1_22:0 (+)**	**−1.1**	**−1.5**	− 1.5 **
**26**	**Cer_NS d18:1_22:1 (+)**	**− 1.0**	**−1.6**	− 1.5 *
**27**	**Cer_NS d18:1_24:0 RT:12.277 (+)**	**− 1.2**	−1.5 *	− 1.4 *
**28**	**Cer_NS d18:1_24:0 RT:13.671 (+)**	**− 1.1**	−1.6 *	− 1.3 *
**29**	**Cer_NS d18:1_24:1 RT:10.678 (+)**	**− 1.0**	−1.5 *	− 1.1 **
**30**	**Cer_NS d18:1_24:1 RT:11.654 (+)**	**− 1.2**	−1.8 **	− 1.7***
**31**	**Cer_NS d18:1_24:1 RT:12.272 (+)**	**− 1.1**	**−1.6**	− 1.4 *
**32**	**Cer_NS d18:1_24:1 (−)**	**−1.0**	− 1.6 *	−1.4 *
**33**	**Cer_NS d18:1_24:2 (+)**	**−1.1**	**−1.8**	− 1.4 *
**34**	**Cer_NS d18:1_24:2 (−)**	**1.1**	**−1.6**	−1.5 *
**35**	**Cer_NS d18:2_18:0 (−)**	**1.0**	−1.2 *	**− 1.1**
**36**	**Cer_NS d18:2_18:0 (+)**	**1.1**	−1.2 *	−1.1 **
**37**	**Cer_NS d18:2_20:0 (−)**	**1.2**	**−1.5**	−1.2 *
**38**	**Cer_NS d18:2_20:0 (+)**	**−1.0**	−1.4 *	− 1.2 *
**39**	**Cer_NS d18:2_22:0 (−)**	**1.4**	**−1.6**	−2.1 *
**40**	**Cer_NS d18:2_22:0 (+)**	**1.2**	**−1.5**	−2.0 *
**41**	**Cer_NS d18:2_24:0 (+)**	**−1.1**	**−1.6**	− 1.4 *
**42**	**Cer_NS d20:1_18:0 (−)**	**1.1**	**−1.4**	−1.3 **
**43**	**Cer_NS d20:1_18:0 (+)**	**−1.1**	**−1.4**	− 1.3***
**44**	**Cer_NS d42:1 (−)**	**−1.1**	**− 1.6**	−1.5 *
**45**	**Cer_NS d42:2 (−)**	**−1.0**	−1.6 *	− 1.4 *
**46**	**Cer_NS d46:1 (−)**	2.0 *	**1.4**	**1.0**
**47**	**SHexCer d18:1_16:0 (+)**	**−1.2**	**−1.0**	1.3 *
**48**	**SHexCer d36:1 (−)**	**1.6**	1.9 *	4.6***
**49**	**SHexCer d40:1 (−)**	**1.3**	**1.1**	2.0***

Not significant

*p = < 0.05.

**p = < 0.01.

***p = < 0.001

**Fig. 5:**
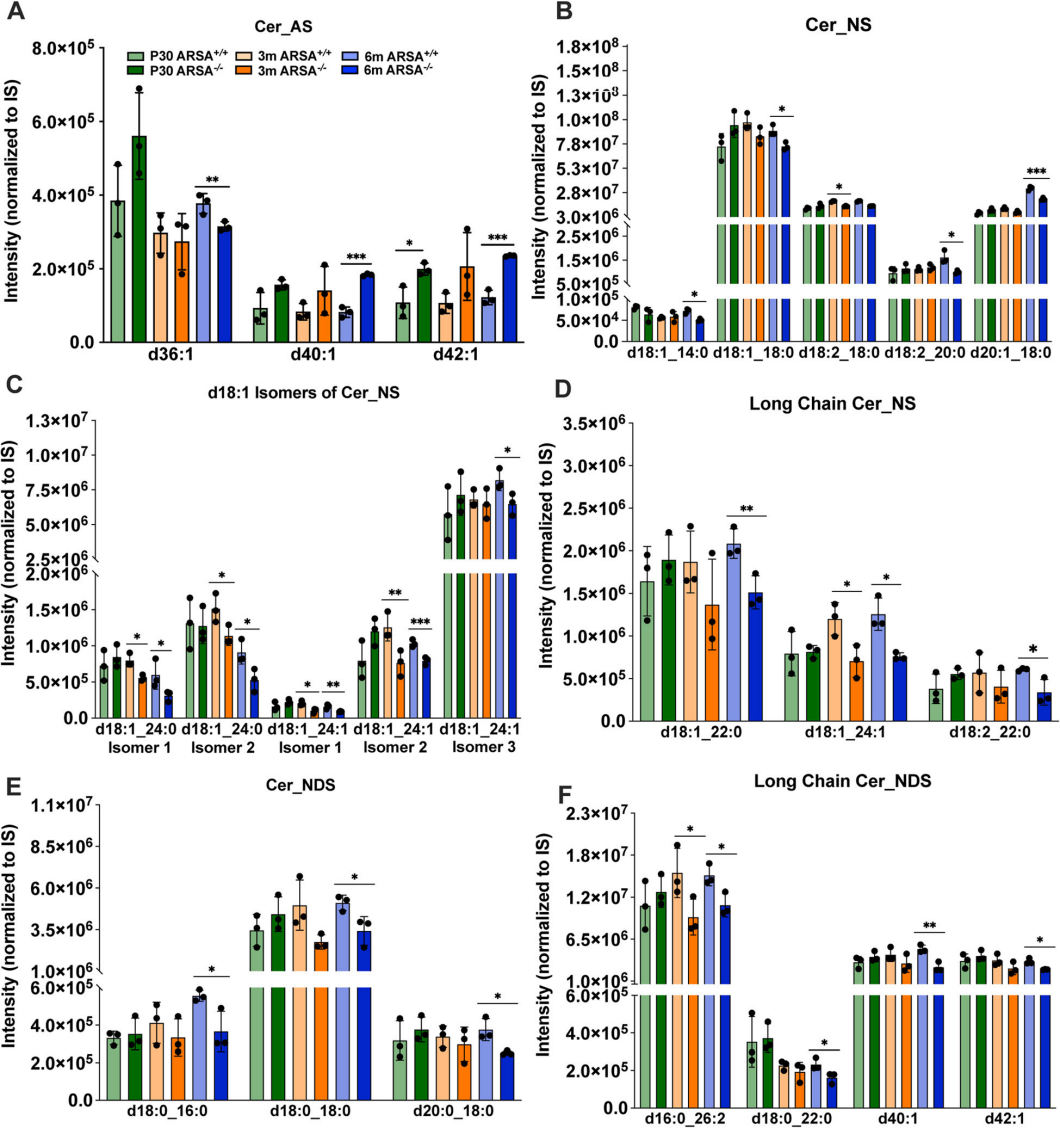
Relative ceramide levels observed in the brain derived EVs from MLD and control mice. Relative levels of ceramides were plotted for **A**) ceramide alpha-hydroxy fatty acid-sphingosine (Cer_AS), **B**) ceramide non-hydroxyfatty acid sphingone (Cer_NS), **C**) d18:1 Cer_NS isomers, **D**) Long chain Cer_NS (> 20 carbons), **E**) ceramide non-hydroxyfatty acid-dihydrosphingosine (Cer_NDS) and **F**) long chain Cer_NS lipid species at 30 postnatal days (P30, green), 3 months (3 m, orange) and 6 months (6 m, blue) of age for control (*ARSA*^*+/+*^) and MLD mice (*ARSA*^*−/−*^). Error bars represent mean ± SEM, n = 3 biological replicates per genotype and time point. Significance was determined via an unpaired t-test (*p* < 0.05, * *p* < 0.01, **; *p* < 0.001, ***)

The relative abundance of hydroxylated and non-hydroxylated forms of ceramides, Cer_AS (Fig. 5A) and Cer_NS (Fig. 5B) was also evaluated. When considering the alpha-hydroxylated form of ceramide, a significant increase for d40:1 and d42:1 in EVs from MLD mice EVs relative to control at 6 m of age was found. In contrast, the opposite trend for the d36:1 species was found at 6 m in MLD EVs relative to control (Fig. 5A**).** When considering the non-hydroxylated ceramide, a significant decrease in Cer_NS d18:1_14:0, d18:1_18:0 and d18:2_20:0 and d20:1–18:0 was measured in MLD EVs EVs relative to control at 6 m (Fig. 5B). Additionally, multiple structural isomers for the d18:1 Cer_NS species were also identified (Fig. 5C). Here, similarly to the other Cer_NS species, significant decreases of d18:1_24:0 (3 m and 6 m) for both isomer 1 and 2 and d18:1_24:1 for isomer 1 and 2 (3 m and 6 m) as well as isomer 3 (6 m) were detected in MLD EVs relative to control (Fig. 5C). Moreover, analysis of Cer_NS lipid species containing long chain acyl groups (> 20 carbons) showed the same trend with a significant decrease of d18:1_22:0 (6 m) and d18:2_22:0 (6 m) in MLD EVs relative to control (Fig. 5D). Numerous non-hydroxylated dihydro ceramide derivatives (Cer_NDS) were also detected. Like the other non-hydroxylated ceramides, significant decreases in d16:0_18:0 (6 m), d18:0_18:0 (6 m) and d20:0_18:0 (6 m) (Fig. 5E) and d16:0_26:2 (3 m and 6 m), d18:0_22:0 (6 m), d40:1 (6 m) and d42:1 (6 m) were measured in MLD EVs relative to control (Fig. 5F), indicating that the dihydro derivative (Cer_NDS) shows the same trend as the Cer_NS species.

**Fig. 6:**
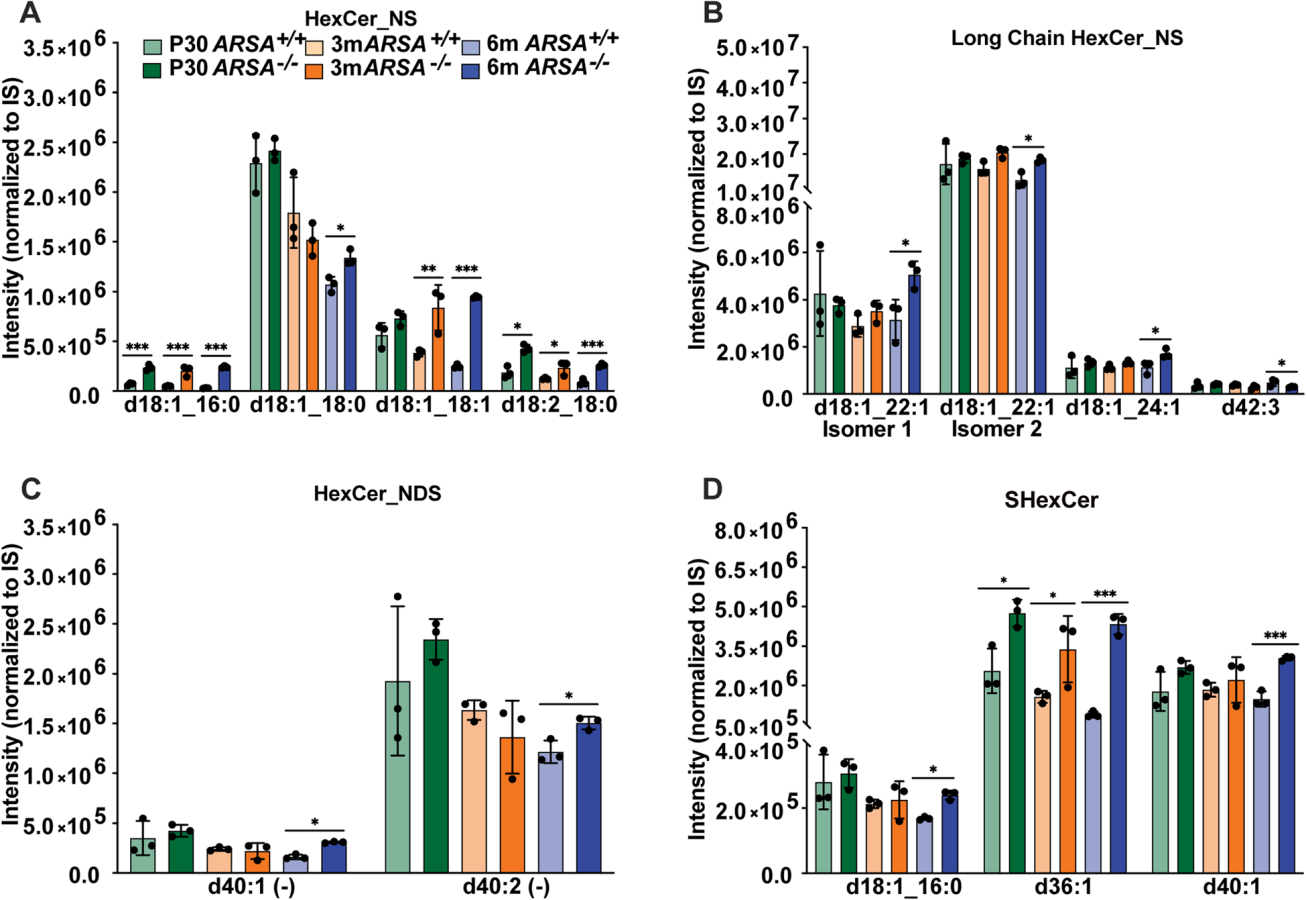
Relative galactosylceramide and sulfatide levels observed in the brain derived EVs from MLD and control mice. Relative levels of galactosylceramide (HexCer) and sulfatides (SHexCer were plotted for **A**) hexosylceramide non- hydroxyfatty acid-phingosine (HexCer_NS), **B**) Long chain HexCer_NS (> 20 carbons), **C**) hexosylceramide non-hydroxyfatty acid-dihydrosphingosine (HexCer_NDS), **D**) sulfatides (SHexCer) at 30 postnatal days (P30, green), 3 months (3 m, orange) and 6 months (6 m, blue) of age for control (*ARSA*^*+/+*^) and MLD (*ARSA*^*−/−*^) mice. Error bars represent mean ± SEM, *n* = 3 biological replicates per genotype and time point. Significance was determined via an unpaired t-test (*p* < 0.05, * *p* < 0.01, **; *p* < 0.001, ***)

Next, we determined the relative levels of hexosylceramides in MLD EVs. Numerous alterations in HexCer_NS (Fig. 6A) and their long chain derivatives (Fig. 6B) were identified, where a significant increase in d18:1_16:0 (P30, 3 m and 6 m), d18:1_18:0 (6 m), d18:1_18:1 (3 m and 6 m) and d18:2_18:0 (P30, 3 m and 6 m), d18:1_22:1 (both isomers 1 and 2, (6 m), d18:1_24:1 (6 m) and d42:3 (6 m) in MLD EVs relative to control. Additionally, we found the same trend for the dihydro HexCer_NS derivatives (HerCer_NDS), where significant increases for d40:1 (6 m) and d40:2 (6 m) were found in MLD EVs (Fig. 6C). Lastly, significant increases in sulfatide species (SHexCer) for d18:1_16:0 (6 m), d36:1 (P30, 3 m and 6 m) and d40:1 (6 m) were found in MLD EVs relative to control. (Fig. 6D). While the MS/MS data was unable to confirm the individual fatty acyl chain compositions, it is possible that these lipids are d18:1_C18:0 and d18:1_20:0 respectively given the common abundance of these fatty acyls.

**Fig. 7:**
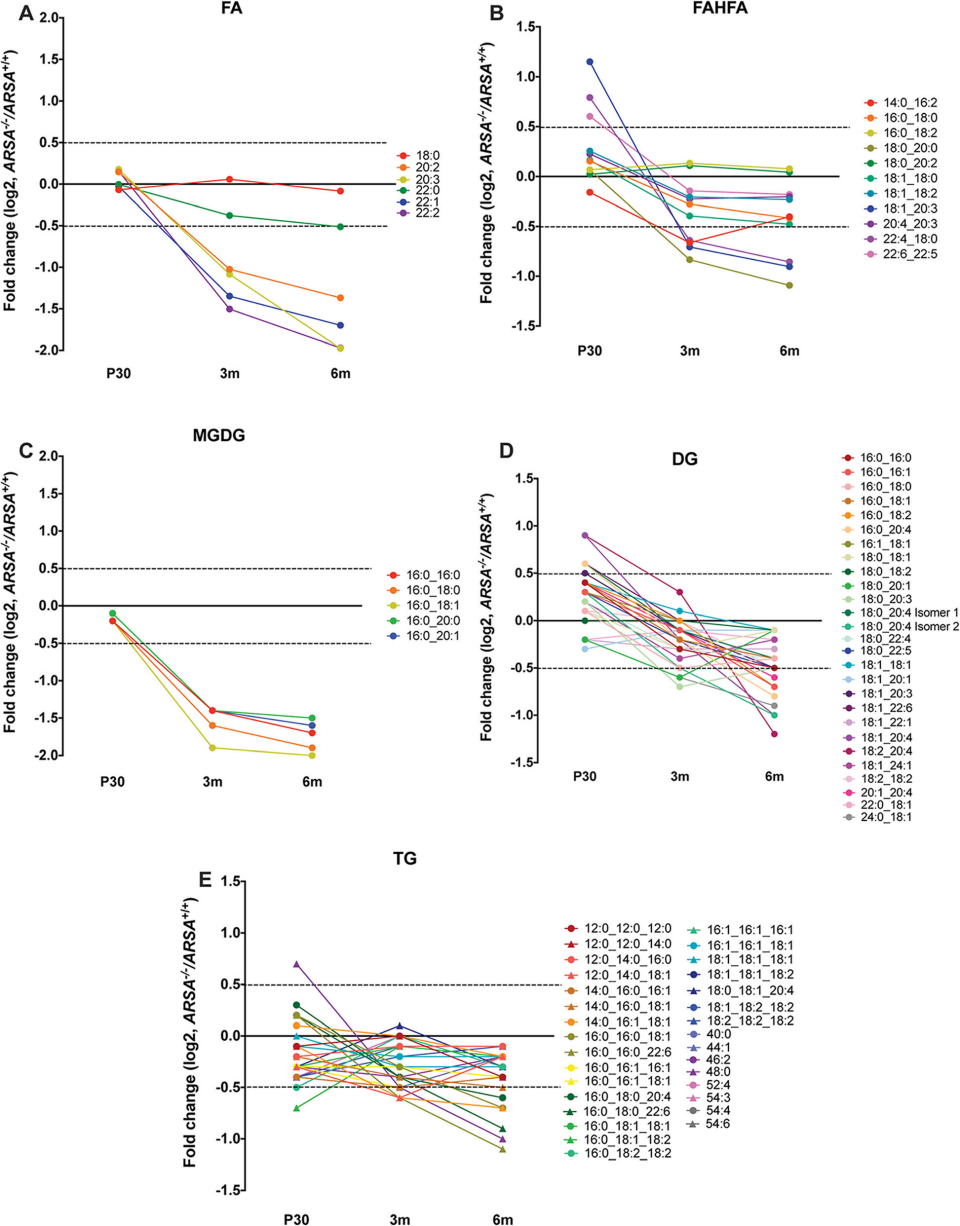
Non-ceramide derived lipid class alterations observed in brain derived EVs from MLD and control mice. Scatter plots for different lipid classes that show a general trend with increasing time points of disease. The average of fold changes plotted for MLD (*ARSA*^*−/−*^) relative to control mice (*ARSA*^*+/+*^*)* at 30 postnatal days (P30), 3 months (3 m) and 6 months (6 m) of age are shown. A downward trend was observed in parallel with disease including **A**) fatty acids (FA), **B**) fatty acid ester of hydroxyl fatty acids, **C**) monogalactosyldiacylglycerols (MGDG), **D**) diacylglycerols (DG) and E) triacylglycerols (TG). *N* = 3 biological replicates per genotype and time point

The untargeted lipidomic analysis of EVs enriched from MLD and control mice revealed numerous, altered lipid species belonging to a broad range of other lipid classes. This includes acylcarnatines (ACar), bis (monoacylglycero) phosphates (BMP)/ phosphatidylglycerols (PG), diacylglycerol (DG), ether-linked phophatidylethanolamines (EtherPE), fatty acids (FA), fatty acid ester of hydroxyl fatty acids (FAHFA), lysophophatidylcholines (LPC), lysophosphatidylethanolamines (LPE), lysophosphatidylinositols (LPI), lysophosphatidylserines (LPS), monogalactosyldiacylglycerols (MGDG), phophatidylcholines (PC), phosphatidylethanolamines (PE), phosphatidylinositols (PI), phosphatidylserines (PS), and triacylglycerols (TG) as well as other ceramide derived lipid species including sphingomyelins (SM). To evaluate whether any of the lipid classes correlate with disease progression, scatter plots were generated (Fig. 7 and additional11). Here, the fold change (MLD relative to control mice) for P30, 3 m and 6 m time point of age were plotted for different lipid classes containing a minimum of two identified lipid species. A downward trend of FA levels was observed in MLD mice, where a greater slope is observed with increasing desaturation of the acyl chain (Fig. 7A). Interestingly, we also found a decrease in numerous FAHFA species with the greatest decrease in species containing C20:0, C20:3 and C20:4 fatty acyl chains (Fig. 7B) that have been reported to exhibit anti-inflammatory effects [36]. Lastly, a downward trend in monogalactosyldiacylglycerol species (MGDG), most pronounced from the P30 to 3 m time points was observed (Fig. 7C). Related acylglycerol lipids showed a similar trend, where numerous DGs (Fig. 7D) and TGs (Fig. 7E) reveal a very pronounced decrease with disease. On the other hand, lipid species including ACars, EtherPEs, EtherPCs, LPCs, LPEs, PCs, PEs, PIs, PGs/BMPs (structural isomers), PSs and SMs revealed numerous alterations of mixed trends (Additional file 11). Taken together, we found that FAs, FAHFAs and acylglycerol lipids are disease-modified lipids, previously uncharacterized in MLD. Our study provides with several new EV-lipid candidates for consideration as biomarkers and potential indicators of disease progression in MLD.

## Discussion

To isolate brain-derived EVs from mouse brain tissue, we adapted the protocol by Perez-Gonzales and colleagues, originally optimized to isolate exosomes from total brains [24]. To date, standardized methods to enrich EVs from brain tissue remains a major challenge in vesicle research. To enrich EVs with a broader range of size, not just exosomes, [15] a 100 μm and subsequent 40 μm filtration step were used. Evaluation of the size range of isolated EVs confirmed the presence of large EVs (20-600 nm). Using our modified enrichment protocol, brain-derived EVs were isolated from MLD and control mice across different time points of the disease. These collection timepoints were selected based on observed characteristics reported in MLD mice [19], where myelination and sulfatides synthesis peak at P30 [37] and at 3 m of age, myelination is established but turn-over is evident [38]. At 6 m of age, MLD mice show clear signs of regional sulfatide storage [19]. Males and females were indiscriminately included in the biological samples since preliminary results did not show any significant sex-dependent difference in EVs numbers.

Overall, our characterization of the number of EVs, protein content and size showed consistent similarities between EVs from both genotypes. It is important to note that in other LSDs such as Gaucher disease, differences in EV size were reported in plasma [39]. However, while small, non-significant, differences were observed in EV sizes between MLD and age matched control mice, these changes seem not to be disease related. Rather, this increase in EV size at 3 m may be related to the peak of brain maturation (3 m) determined for rodents [21], and the possible functional role(s) of EVs in postnatal development. Overall, the consistent finding of similar vesicular fractions at each time point and for both genotypes suggest a tight control of the production of EVs in the brain. This may represent their role in regulating homeostasis of endo/lysosome and plasma membrane trafficking [40].

Lipidomic analysis revealed numerous altered lipid species in MLD EVs. It is well known that sulfatide lipids accumulate in MLD due to the lack of ARSA expression; however, this is the first report confirming their accumulation in brain-derived EVs. Many of the altered sulfatides correspond to those containing an 18-carbon fatty acyl chain (including 1 and 2 double bonds), with a minor presence of a 22-carbon fatty acyl chain (encompassing 1 double bond). Sulfatides with 18-carbon fatty acyl chains have been previously reported as the most abundant lipids in MLD mice [41], with a 4-fold increase in brain tissues [42]. These observations support previous reports that short chain (≤18 carbons) fatty acyl sulfatides may be associated with EVs [41]. Our current finding of a significant increase of sulfatides containing short-chain fatty acyl chains in MLD mice might indicate that these sulfatide are arising from neurons and/or astrocytes and consistent with previous reports [41] .

The presence of increased levels of sulfatides in MLD EVs may reflect a compensation mechanism by which affected neural cells remove accumulated sulfatides from cell membranes by exocytosis to reduce toxic levels. It may also represent a component in the overall mechanism of pathogenesis in MLD, as a way to spread disease to neighboring cells, which normally do not accumulate sulfatides to toxic levels. Determining the extent to which these hypotheses hold true requires specific cell biological approaches which, while currently in progress in our laboratory, are beyond the scope of this particular report. While some studies have shown that amyloid proteins can spread disease via EVs [43–48], whether the same applies to sphingolipids such as sulfatides remains uncertain. To date, few studies have addressed the role of brain-derived EVs in sphingolipidoses [18]. Sphingolipidoses appear to have enhanced exosome secretion, presumably due to the increased accumulation of non-degraded products in the lysosomes and/or an increase in the production of microvesicles [14, 49, 50]. Thus, understanding this aspect of EV biology is important not only to elucidate their contribution to MLD, but also in other disease processes [11, 15, 51–55].

This initial study demonstrates that there is mobilization of sulfatides in MLD EVs. Additionally, ceramides which are precursors to sulfatides, were significantly altered in MLD EVs, with a general downregulation of Cer_NS and Cer_NDS, including both short and long chain fatty acyl chains, but we observed a significant upregulation of Cer_AS, detected at 3 m and 6 m of age. Considering that there is no difference in trend for non-hydroxylated ceramide lipids containing long chains compared to shorter chains (i.e., 16–20 carbons) between genotype, it does not appear that these alterations in MLD EVs are directly related to chain length. However, given the opposite trend found between the hydroxylated and non-hydroxylated forms, these data may suggest that alpha-hydroxylation of the ceramides might play a role in MLD pathology, although further experiments are needed to better understand this observation. Additionally, the increase in Cer_AS along with a decrease in Cer_NS and Cer_NSD are intriguing. Whether these findings reflect the lipidomic profile of the cells they are coming from or a change that confers membrane rigidity [56] needs to be addressed. Evaluation of neural cell markers of enriched EVs did not provide a correlate between lipid changes in EVs and cell types responsible for these observations. Thus, while the possibility that EVs are contributing to pathogenic processes in neighboring cells remains high, additional studies are needed to determine the specific pathological roles of sulfatides and/or any of the other lipids identified in this study when secreted via EVs.

Our lipidomic analysis also revealed additional changes in non-ceramide derived lipid species. Various PG (e.g. PG 16:0_18:0 or PG 20:4_20:4) increased in MLD EVs respect to control during disease while some BMP species (e.g. BMP 18:1_22:6 or BMP 20:4_22:6, Fig. S7 H) decreased in older MLD mice. These changes are consistent with the finding of increased PG and BMP (18:1/22:6) in urine and skin fibroblasts from human juvenile and adult forms of MLD [57] and underlines the possibility that some of these lipids may also be considered for biomarking disease progression. Moreover, LPC species in MLD EVs from 3 m and 6 m were observed to be elevated. LPC species are known lipids that promote demyelination and inflammation [58, 59], and their presence in secreted EVs underlines a possible role in the pathogenic mechanisms of myelin damage. ACars isoforms were also increased in MLD EVs, specifically at 6 m of age. These ACars are found altered in conditions with mitochondrial dysfunction and/or fatty acid overload [60]. In contrast, some glycerophospholipids, such as PE, PS, PI and PC, were decreased in MLD EVs respect to control. Furthermore, FAs and FAHFAs progressively decreased their content in the mutant EVs. The significance of these latter changes is still uncertain. FAs have been implicated in neural cell pathology in LSDs [61], while little is known regarding the FAHFAs, except their potential role in anti-inflammatory effects [36]. Acylglycerol lipids are commonly hydrolyzed to yield *FA* for energy or undergo re-esterification for the storage of FAs. Given that FA levels were decreased in MLD EVs, acylglycerols may be continually hydrolyzed to compensate for the low levels of FAs in MLD mice.

### Strengths and limitations

Our lipidomic analysis provides for the first time a quantitative insight into the lipid signature and composition of brain-derived EVs isolated from MLD mice. As expected, our analysis confirmed the presence and elevation of sulfatides in MLD EVs, and identified additional and previously uncharacterized lipid fluctuations in MLD. The results of this study contribute to better understand the pathogenesis of MLD and provide with new candidates for biomarking disease progression and/or therapy follow-up. Our modified protocol used to isolate EVs resulted in highly reproducible preparations without high levels of contamination (i.e., early endosomal, synaptic vesicles, endoplasmic reticulum membranes). However, the major caveat of this protocol is the heterogeneity of vesicles expressing markers from neurons, oligodendrocytes, astrocytes and microglial cells. Because this enrichment protocol uses structural and buoyancy properties for separation, the observed heterogeneity of EVs indicates similar physicochemical properties of EVs regardless their cell of origin, which impedes refined separation based on cell lineages. Further separation of EVs according to cellular origin might require the use of tagging systems and subsequent tag-mediated separations. Additionally, the presence of isobaric species is a challenge with mass spectrometry-based analysis. For example, the lipidomic analysis used in this study was not able to discriminate which hexose moiety was present on identified HexCer lipids, namely glucose or galactose. Lastly, because the MLD mouse model does not fully recapitulate demyelination as observed in the human infantile forms of MLD [20], more studies using human-derived samples would be needed. For example, previous studies have shown that EVs from the brain can be found mobilized to biofluids such as plasma or cerebrospinal fluid [62, 63]. Thus it will be of interest to study if similar lipid profiles found using the MLD mouse model also translate to affected humans. If such is the case, some of the lipids identified in our study may become biomarkers to use in MLD clinical follow-up and therapeutic studies.

## Conclusions

In summary, our lipidomic analysis confirmed that sulfatides are secreted and likely mobilized via EVs in the brain of MLD mice, along with sulfatide precursors (ceramides), and coincides with previous observations of sulfatide accumulation in MLD. Additionally, we identified numerous FA species and their branch chain derivatives as well as lipids known to store fatty acids with diminished levels in MLD EVs and propose that FAs, FAHFAs and acylglycerol lipids may be relevant candidates for biomarkers of MLD disease.

## Supplementary Information

**Additional file 1.** List of antibodies used for Western blot studies.

**Additional file 2. **Densitometry analysis of western blots. EV samples were subjected to electrophoresis and Western blot analysis for common cellular vesicle markers (A) and cell type markers (B-I). Densitometric analysis of western blots presented on Fig. 1 were measured utilizing ImageJ software for: programmed cell death 6-interacting protein (ALIX), flotillin 1 (FLOT1), early endosome antigen 1 (EEA1), endoplasmic reticulum calnexin (CALX) and synaptic synaptotagmin 1 protein (SYT1) utilizing Rab5B as housekeeping gene (*n* = 1 per genotype and time point). Cell type specific markers for: glial fibrillary acidic protein (GFAP, astrocytes), beta-tubulin III (TUBB3, neurons), myelin basic protein (MBP, oligodendrocytes), allograft inflammatory factor 1 (IBA1, microglia) and MHCII. Also, reactivity for Actin and sodium/potassium-transporting ATPase subunit beta-1 (Na + K+ ATPase) and Rab5B utilizing Actin as housekeeping gene. Relative abundance of Actin between samples it is shown (n = 1 per genotype and time point).

**Additional file 3.** Particle size distribution of isolated Extracellular vesicles. Representative histograms of particle concentration for isolated extracellular vesicles isolated from control (*ARSA*^*+/+*^) and MLD (*ARSA*^*−/−*^*)*murine brain tissue obtained from postnatal day 30 (P30), 3 months (3 m) and 6 months (6 m) of age. Concentration is measured as number of particles per milliliter of solution. Black lines represent the average and red lines the range of all data recorded.

**Additional file 4.** Summary of EVs size (mean and mode).

**Additional file 5. **Particle size distribution of isolated Extracellular vesicles after a density gradient centrifugation. Representative histograms of particle concentration for isolated extracellular vesicles isolated from control (*ARSA*^*+/+*^*)*and MLD (*ARSA*^*−/−*^) murine brain tissue corresponding to a pool of fractions B, C and D (Fig. 1) obtained after the sucrose gradient centrifugation from postnatal day 30 (P30), 3 months (3 m) and 6 months (6 m) of age. Concentration is measured as number of particles per milliliter of solution. Black lines represent the average and red lines the range of all data recorded.

**Additional file 6. **Extracellular particle size distribution for fraction B,C and D of postnatal day 30 mice. Representative histograms of particle concentration for isolated extracellular vesicles isolated from control *(ARSA*^*+/+*^*)* and MLD *(ARSA*^*−/−*^*)* murine brain tissue. Concentration is measured as number of particles per milliliter of solution for obtained from postnatal day 30 (P30) mice in fractions B, C and D (Fig. 1). Black lines represent the average and red lines the range of all data recorded.

**Additional file 7.** Lipids identified via MS/MS fragmentation in positive and negative ion modes.

**Additional file 8.** Significant altered lipids identified via MS/MS fragmentation in positive and negative ion modes.

**Additional file 9. **Heatmap representation of the intensity for lipids measured in brain derived EVs from MLD and control mice. Lipid species identified from the liquid chromatography mass spectrometry analysis of extracellular vesicles (EVs) for (A) acylcarnatine (ACar), (B) bis (monoacylglycero) phosphate (BMP)/ phosphatidylglycerol (PG), (C) ceramide non-hydroxyfatty acid-sphingosine (Cer_NS), (D) ceramide non-hydroxyfatty acid-dihydrosphingosine (Cer_NDS), (E) ceramide alpha-hydroxy fatty acid-sphingosine (Cer_AS), (F) ceramide esterified omega-hydroxy fatty acid-dihydrosphingosine (Cer_EODS), (G) (DG) diacylglycerol, (F) ether-linked phophatidylethanolamine (EtherPE), (G) fatty acid (FA), (H) fatty acid ester of hydroxyl fatty acid (FAHFA), (I) hexosylceramide non-hydroxyfatty acid-sphingosine (HexCer_NS), (J) hexosylceramide non-hydroxyfatty acid-dihydrosphingosine (HexCer_NDS), (K) Sulfatide (SHexCer), (L) lysophophatidylcholine (LPC), (M) lysophosphatidylethanolamine (LPE), (N) lysophosphatidylinositol (LPI), (O) lysophosphatidylserine (LPS), (P) monogalactosyldiacylglycerol (MGDG), (Q) phophatidylcholine (PC), (R) phatidylethanolamine (PE), (S) phosphatidylethanol (PEtOH), (T) phatidylinositol (PI), (U) phosphatidylserine (PS), (V) sphingomyelin (SM), (W) triacylglycerol (TG), (X) phosphatidic acid (PA) and (Y) cardiolipin (CL) lipid classes are shown for each biological replicate for control *(ARSA*^*+/+*^*)* and MLD *(ARSA*^*−/−*^*)* mice. Lipids noted with (+) or (−) were identified by fragmentation matching in positive or negative ion modes, respectively.

**Additional file 10. **Plots showing the number of altered lipids per class measured in brain derived lipids enriched from MLD and control mice. Bar charts showing the number of lipid species significantly (*p* < 0.05) altered lipid species identified in extracellular vesicle (EV) lipid extracts measured via mass spectrometry analysis in (A) positive and (B) negative ion modes for acylcarnatine (ACar), bis (monoacylglycero) phosphate (BMP)/ phosphatidylglycerol (PG), ceramide non-hydroxyfatty acid-sphingosine (Cer_NS), ceramide non-hydroxyfatty acid-dihydrosphingosine (Cer_NDS), ceramide alpha-hydroxy fatty acid-sphingosine (Cer_AS), diacylglycerol (DG), ether-linked phophatidylethanolamine (EtherPE), fatty acid (FA), fatty acid ester of hydroxyl fatty acid (FAHFA), hexosylceramide non-hydroxyfatty acid-sphingosine (HexCer_NS), hexosylceramide non-hydroxyfatty acid-dihydrosphingosine (HexCer_NDS), sulfatide (SHexCer), lysophophatidylcholine (LPC), lysophosphatidylethanolamine (LPE), lysophosphatidylinositol (LPI), lysophosphatidylserine (LPS), monogalactosyldiacylglycerol (MGDG), phophatidylcholine (PC), phosphatidylethanolamine (PE), phosphatidylinositol (PI), phosphatidylserine (PS), sphingomyelin (SM) and triacylglycerol (TG) lipid classes at postnatal day 30 (P30), 3 months (3 m) and 6 months (6 m) of age for control (*ARSA*^*+/+*^*)* and MLD (*ARSA*^*−/−*^*)* mice.

**Additional file 11. **Derived lipid class that don’t show a correlation across time points of disease in MLD mice. Scatter plots for lipid classes that do not show a correlation across time points of disease in MLD mice. Fold change plotted for MLD *(ARSA*^*−/−*^*)* relative to control *(ARSA*^*+/+*^*)* mice at 30 postnatal days (P30), 3 months (3 m) and 6 months (6 m) of age for (A) acylcarnitine (ACar), (B) ether-linked phophatidylethanolamine (EtherPE), (C) ether-linked phophatidylphophatidylcholine (EtherPC), (D) lysophophatidylcholine (LPC), (E) lysophosphatidylethanolamine (LPE), (F) phophatidylcholine (PC), (G) phosphatidylethanolamine (PE), (H) phosphatidylinositol (PI), (I) phosphatidylglycerol (PG)/ bis (monoacylglycero) phosphate (BMP), (J) phosphatidylserine (PS) and (K) sphingomyelin (SM) lipid classes.

## Data Availability

All raw mass spectrometry data is publicity available at: ftp://massive.ucsd.edu/MSV000086647/.

## Abbreviations

ACar: Acylcarnitine
ADAM10: Metalloproteinase domain-containing protein 10
ALIX: Programmed cell death 6-interacting protein
ARSA: Aryl-sulfatase A
ATP1B1: Sodium/potassium-transporting ATPase subunit beta-1
BCA: Bicinchoninic acid assay
BMP: Bis (monoacylglycero)phosphates
CALX: Calnexin
Cer: Ceramides
Cer_AS: Ceramide alpha-hydroxy fatty acid-sphingosine
Cer_NDS: Ceramide non-hydroxyfatty acid-dihydrosphingosine
Cer_NS: Ceramide non-hydroxyfatty acid-sphingosine
CNS: Central nervous system
DG: Phosphatidylglycerol
ECL: Chemiluminescent reagent
EEA1: Early endosome antigen 1
EtherPE: Ether-linked phophatidylethanolamine
EV: Extracellular vesicle
FA: Fatty acid
FAHFA: Fatty acid ester of hydroxyl fatty acid
FLOT1: Flotillin 1
GALC: Galactocylceramidase or beta-galactocerebosidase
GFAP: Glial fibrillary acidic protein
HexCer: Hexosylceramides
HexCer_AS: Hexosylceramide alpha-hydroxy fatty acid-sphingosine
HexCer_NDS: Hexosylceramide non-hydroxyfatty acid-dihydrosphingosine
HexCer_NS: Hexosylceramide non-hydroxyfatty acid-sphingosine
IBA1: Allograft inflammatory factor 1
LPC: Lysophophatidylcholines
LPE: Lysophosphatidylethanolamines
LPS: Lysophosphatidylserines
LSD: Lysosomal storage disease
m: Month
MBP: Myelin basic protein
MGDG: Monogalactosyldiacylglycerols
MHCII: Major antigen presenting class II
MLD: Metachromatic leukodystrophy disease
NTA: Nanoparticle Tracking Analysis
P: Postnatal day
PC: Phophatidylcholines
PDGFrα: Platelet derived growth factor alpha.
PE: Phosphatidylethanolamines
PG: Phosphatidylglycerol
PI: Phosphatidylinositols
PS: Phosphatidylserines
PNS: Peripheral nervous system
PVDF: Polyvinylidene fluoride
RAB5B: Ras-related protein Rab-5B
SHexCer: Sulfatides
SM: Sphingomyelin
SYT1: Synaptotagmin 1
TBS: Tris buffered saline
TG: Triacylglycerols
TSG101: Tumor susceptibility gene 101 protein
TUBB3: Beta-tubulin III
UIC: University of Illinois at Chicago
