# Supplementary material for: Lipidomic analysis identifies age-disease-related changes and potential new biomarkers in brain-derived extracellular vesicles from metachromatic leukodystrophy mice
Source: Lipids Health Dis. 2022 Mar 27;21:32. doi: 10.1186/s12944-022-01644-8 (PMC8962106; doi:10.1186/s12944-022-01644-8)
Supplement: Supplementary file 1 — Additional file 1. List of antibodies used for Western blot studies. [file 12944_2022_1644_MOESM1_ESM.pdf]

Primary Antibody	Dilution	Host	Vender	Product Number
Anti-ADAM10	1:1000	Rabbit	Chemicon International	AB-19026
Anti-ACTIN	1:1000	Rabbit	Sigma	SAB4301137
Anti-ALIX	1:300	Mouse	EMD Millipore Corp	MABS1277
Anti-AT1BA	1:500	Mouse	EMD Millipore Corp	05-369
Anti-CALX	1:500	Rabbit	Cell Signaling	2679P
Anti-CD74	1:500	Rat	AbD Serotec	MCA46G
Anti-EEA1	1:500	Rabbit	Cell Signaling Technology	3288P
Anti-FLOT1	1:500	Mouse	BD	610820
Anti-GFAP	1:500	Mouse	EMD Millipore Corp	MAB3402
Anti-IBA1	1:500	Rabbit	Invitrogen	PA5-27436
Anti-MBP	1:500	Rabbit	Gift from Dr. Bongarzone	From Dr. Campagnoni's lab
Anti-RA5B	1:300	Rabbit	Santa Cruz Biotechnology	SC-598
Anti-SYT1	1:1000	Mouse	Chemicon International	MAB5200

Anti-TUBB3	1:500	Mouse	Cell Signaling Technology	4466S
**Secondary Antibody**	**Dilution**	**Host**	**Vender**	**Product Number**
Anti-Rabbit, IgG	1:1000	Goat	Cell Signaling	7074S
Anti-Mouse IgG	1:1000	Horse	Cell Signaling	7076S
Anti-Rat IgG	1:10,000	Goat	Jackson Immuno Research	112-035-003
