# Supplementary figures and images for: Lipidomic analysis identifies age-disease-related changes and potential new biomarkers in brain-derived extracellular vesicles from metachromatic leukodystrophy mice
Source: Lipids Health Dis. 2022 Mar 27;21:32. doi: 10.1186/s12944-022-01644-8 (PMC8962106; doi:10.1186/s12944-022-01644-8)

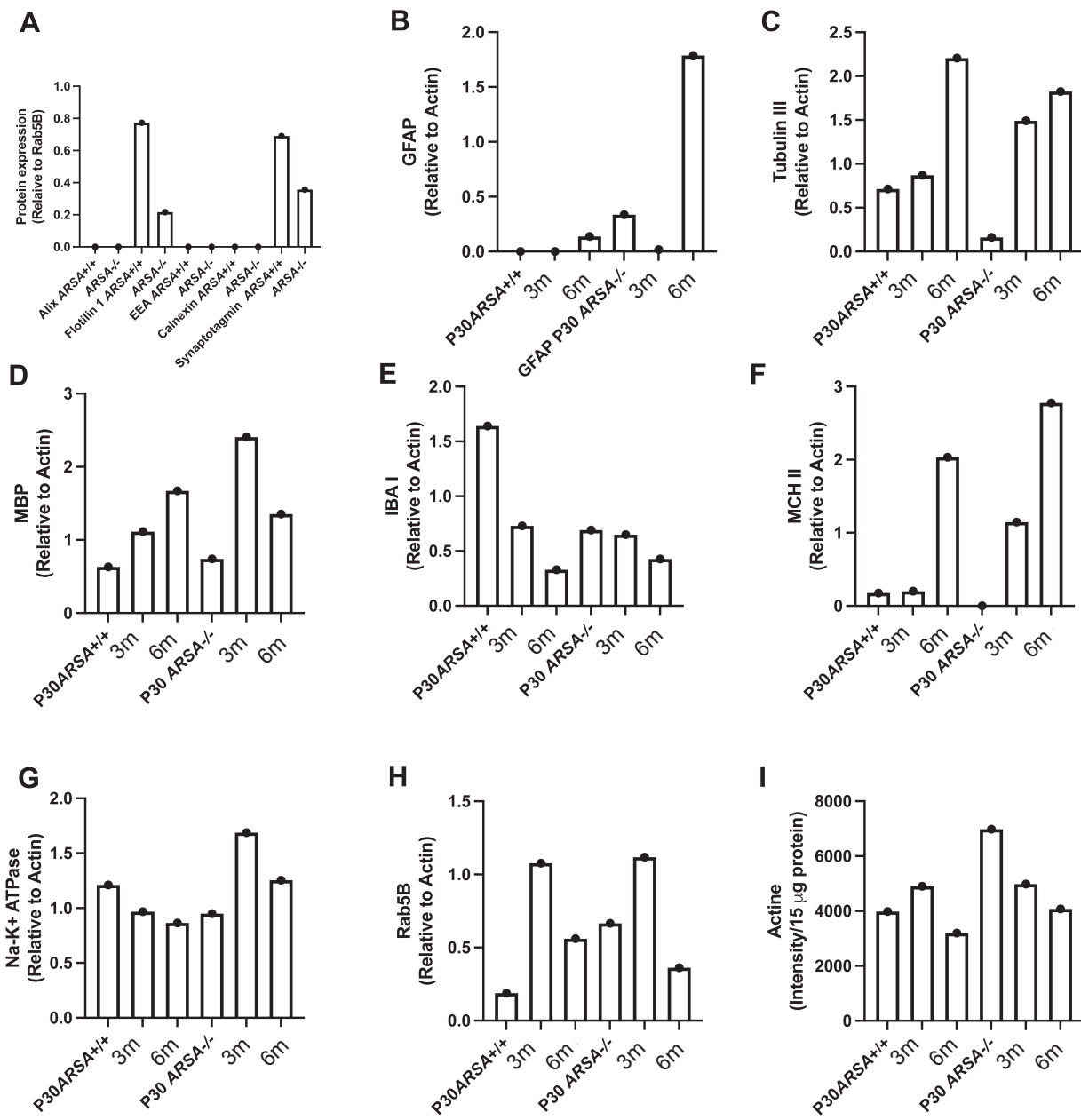

Supplement: Supplementary file 2 — Additional file 2. Densitometry analysis of western blots. EV samples were subjected to electrophoresis and Western blot analysis for common cellular vesicle markers (A) and cell type markers (B-I). Densitometric analysis of western blots presented on Fig. 1 were measured utilizing ImageJ software for: programmed cell death 6-interacting protein (ALIX), flotillin 1 (FLOT1), early endosome antigen 1 (EEA1), endoplasmic reticulum calnexin (CALX) and synaptic synaptotagmin 1 protein (SYT1) utilizing Rab5B as housekeeping gene (n = 1 per genotype and time point). Cell type specific markers for: glial fibrillary acidic protein (GFAP, astrocytes), beta-tubulin III (TUBB3, neurons), myelin basic protein (MBP, oligodendrocytes), allograft inflammatory factor 1 (IBA1, microglia) and MHCII. Also, reactivity for Actin and sodium/potassium-transporting ATPase subunit beta-1 (Na + K+ ATPase) and Rab5B utilizing Actin as housekeeping gene. Relative abundance of Actin between samples it is shown (n = 1 per genotype and time point). [file 12944_2022_1644_MOESM2_ESM.pdf]

ASA+/+

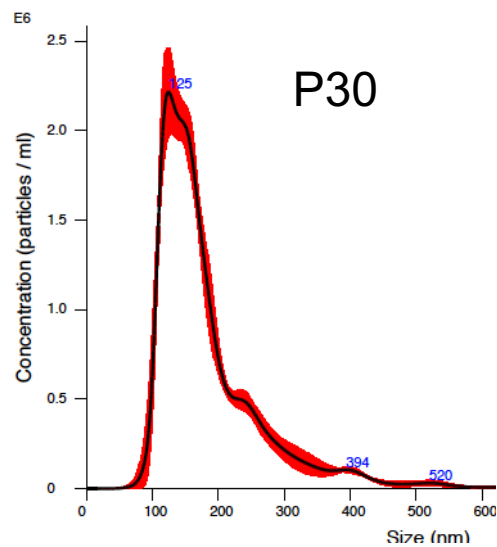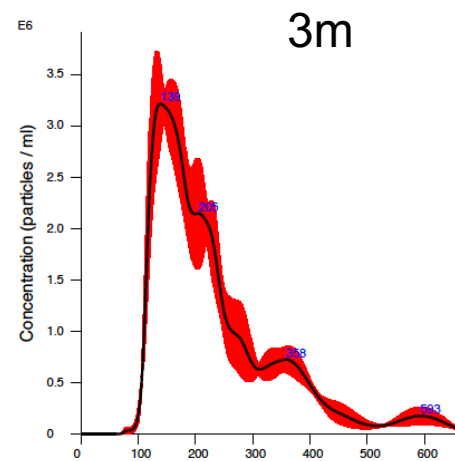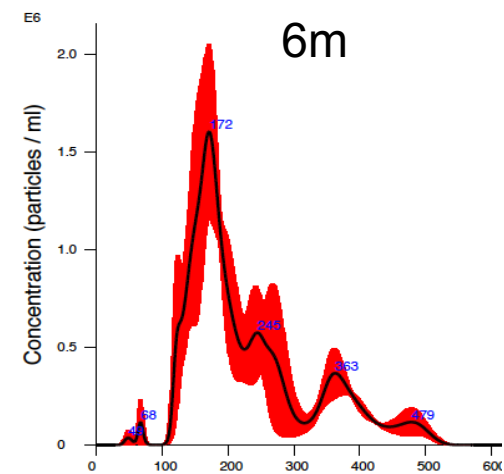

ASA-/-

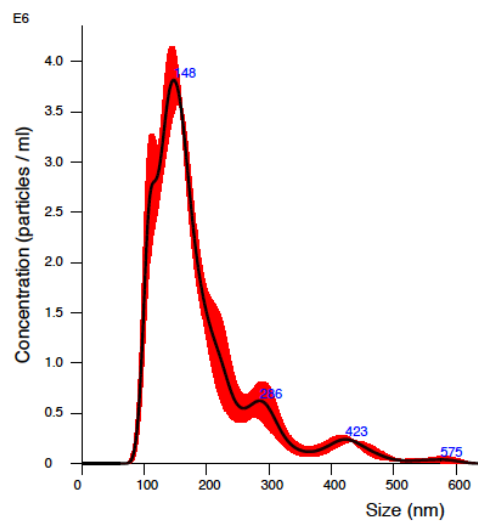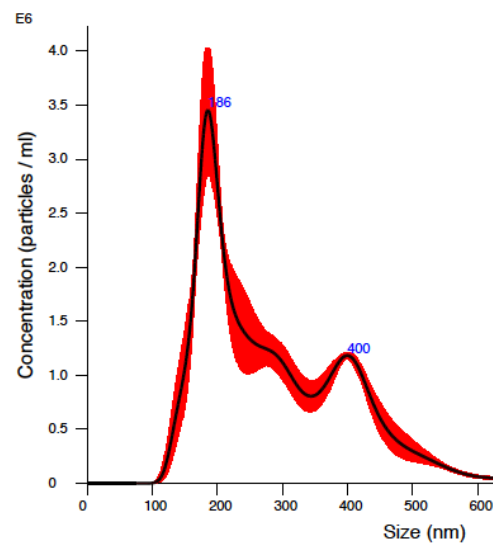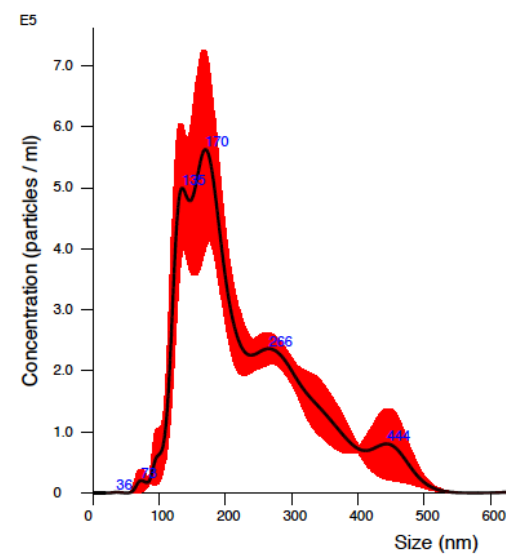

Supplement: Supplementary file 3 — Additional file 3. Particle size distribution of isolated Extracellular vesicles. Representative histograms of particle concentration for isolated extracellular vesicles isolated from control (ARSA+/+) and MLD (ARSA−/−)murine brain tissue obtained from postnatal day 30 (P30), 3 months (3 m) and 6 months (6 m) of age. Concentration is measured as number of particles per milliliter of solution. Black lines represent the average and red lines the range of all data recorded. [file 12944_2022_1644_MOESM3_ESM.pdf]

ASA+/-

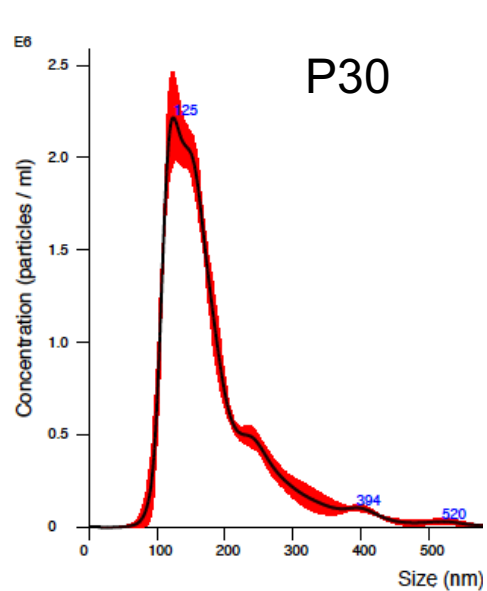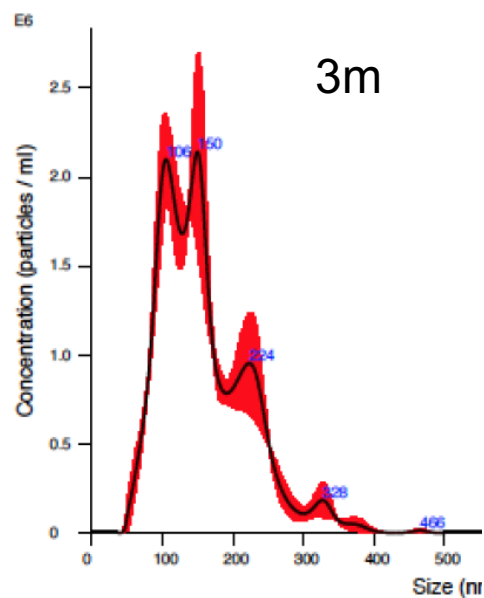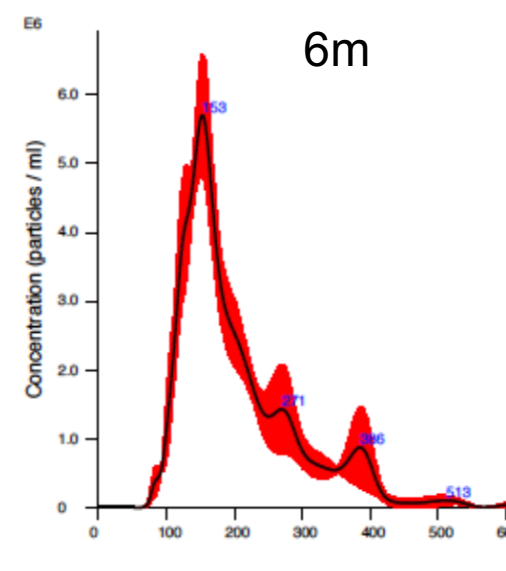

ASA-/-

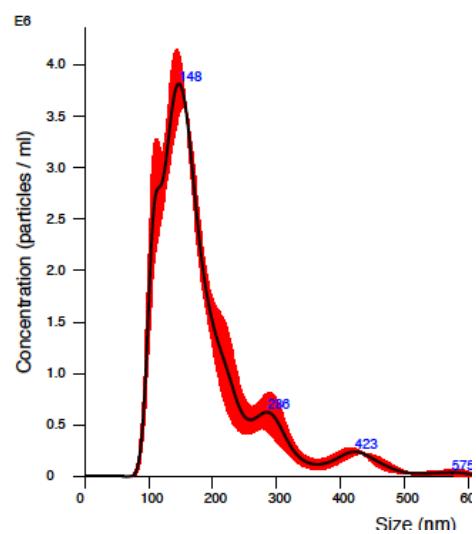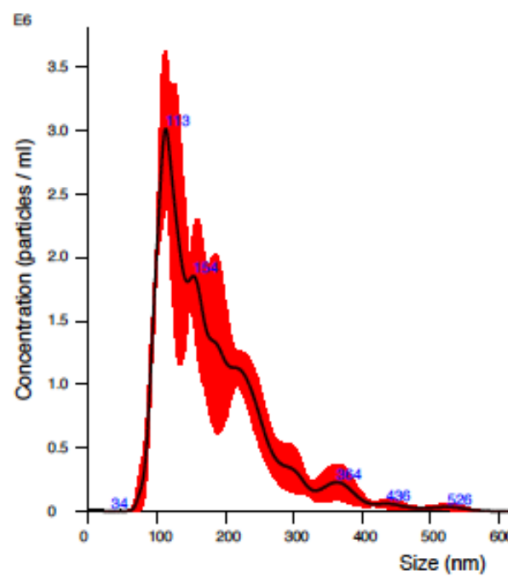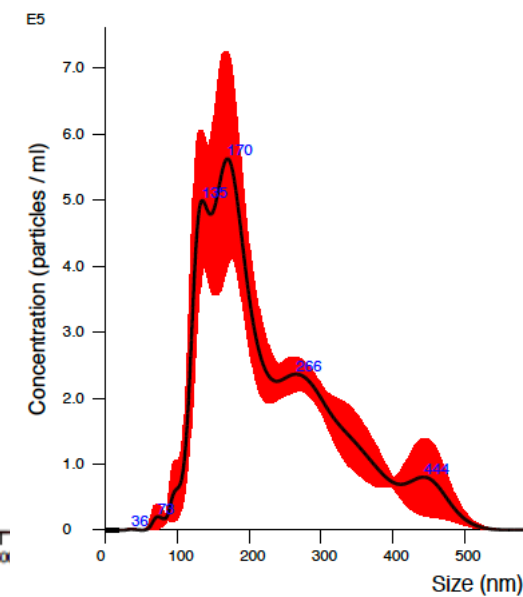

Supplement: Supplementary file 5 — Additional file 5. Particle size distribution of isolated Extracellular vesicles after a density gradient centrifugation. Representative histograms of particle concentration for isolated extracellular vesicles isolated from control (ARSA+/+)and MLD (ARSA−/−) murine brain tissue corresponding to a pool of fractions B, C and D (Fig. 1) obtained after the sucrose gradient centrifugation from postnatal day 30 (P30), 3 months (3 m) and 6 months (6 m) of age. Concentration is measured as number of particles per milliliter of solution. Black lines represent the average and red lines the range of all data recorded. [file 12944_2022_1644_MOESM5_ESM.pdf]

***P30 ARSA<sup>+/+</sup>***

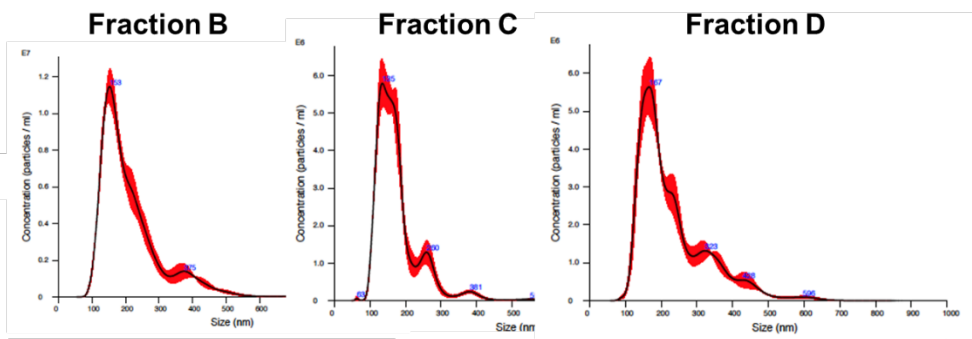

***P30 ARSA<sup>-/-</sup>***

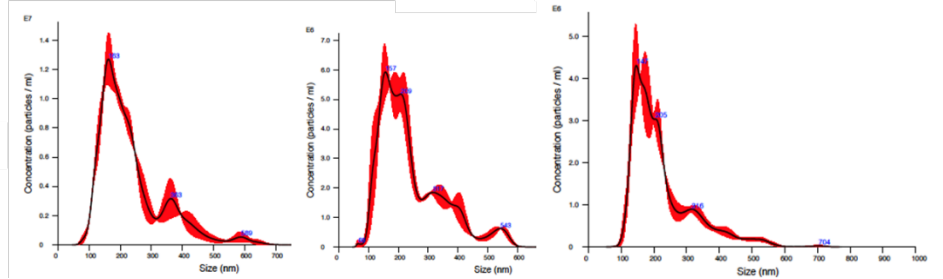

Supplement: Supplementary file 6 — Additional file 6. Extracellular particle size distribution for fraction B,C and D of postnatal day 30 mice. Representative histograms of particle concentration for isolated extracellular vesicles isolated from control (ARSA+/+) and MLD (ARSA−/−) murine brain tissue. Concentration is measured as number of particles per milliliter of solution for obtained from postnatal day 30 (P30) mice in fractions B, C and D (Fig. 1). Black lines represent the average and red lines the range of all data recorded. [file 12944_2022_1644_MOESM6_ESM.pdf]

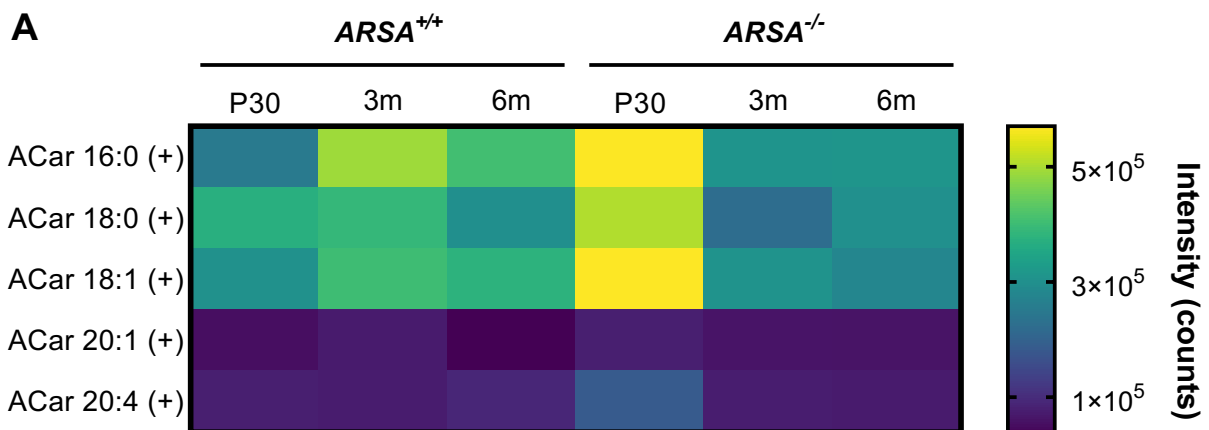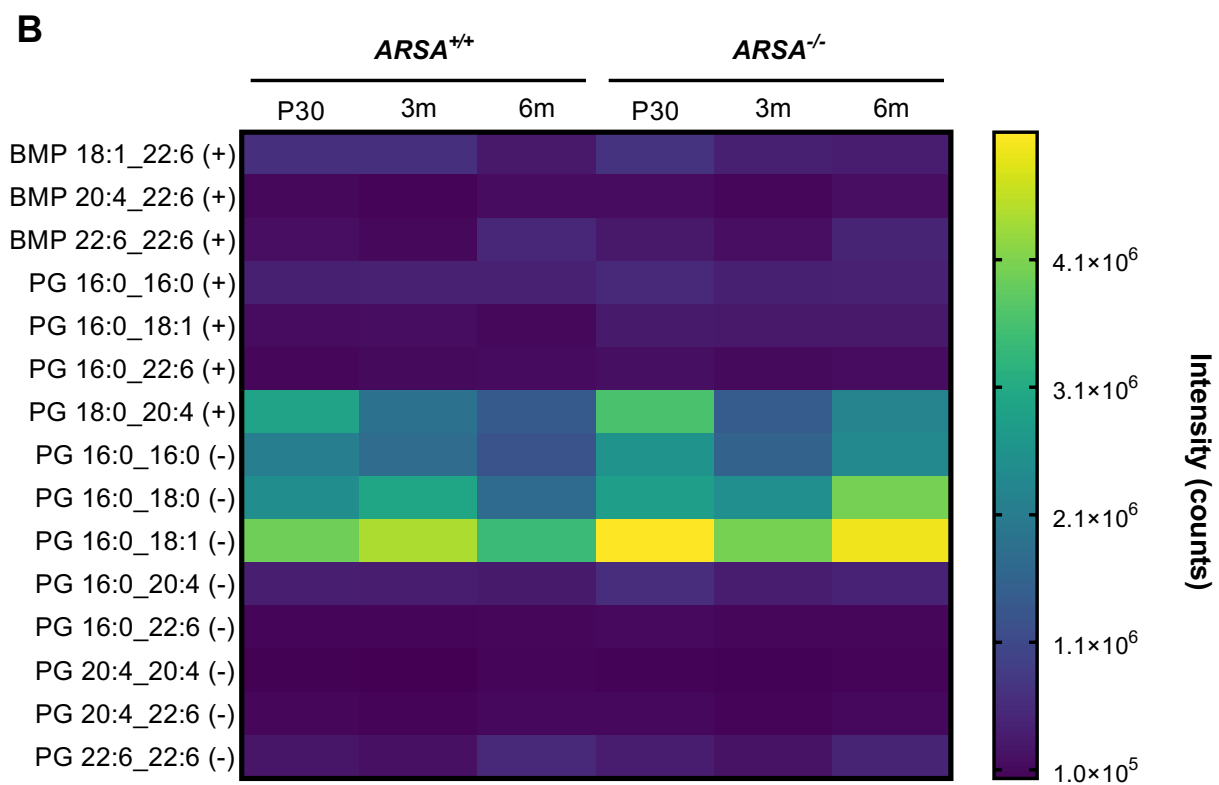

C

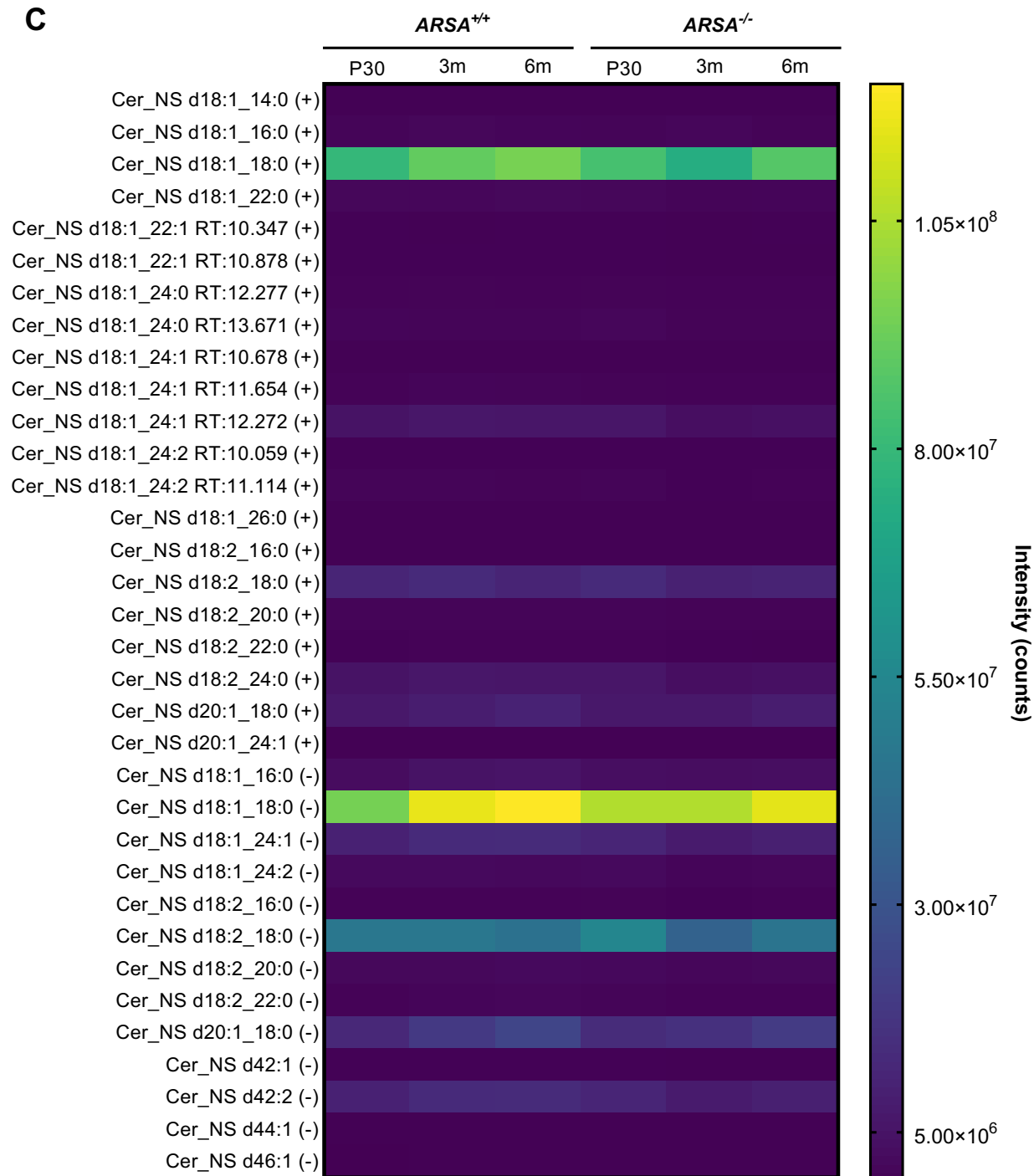

D

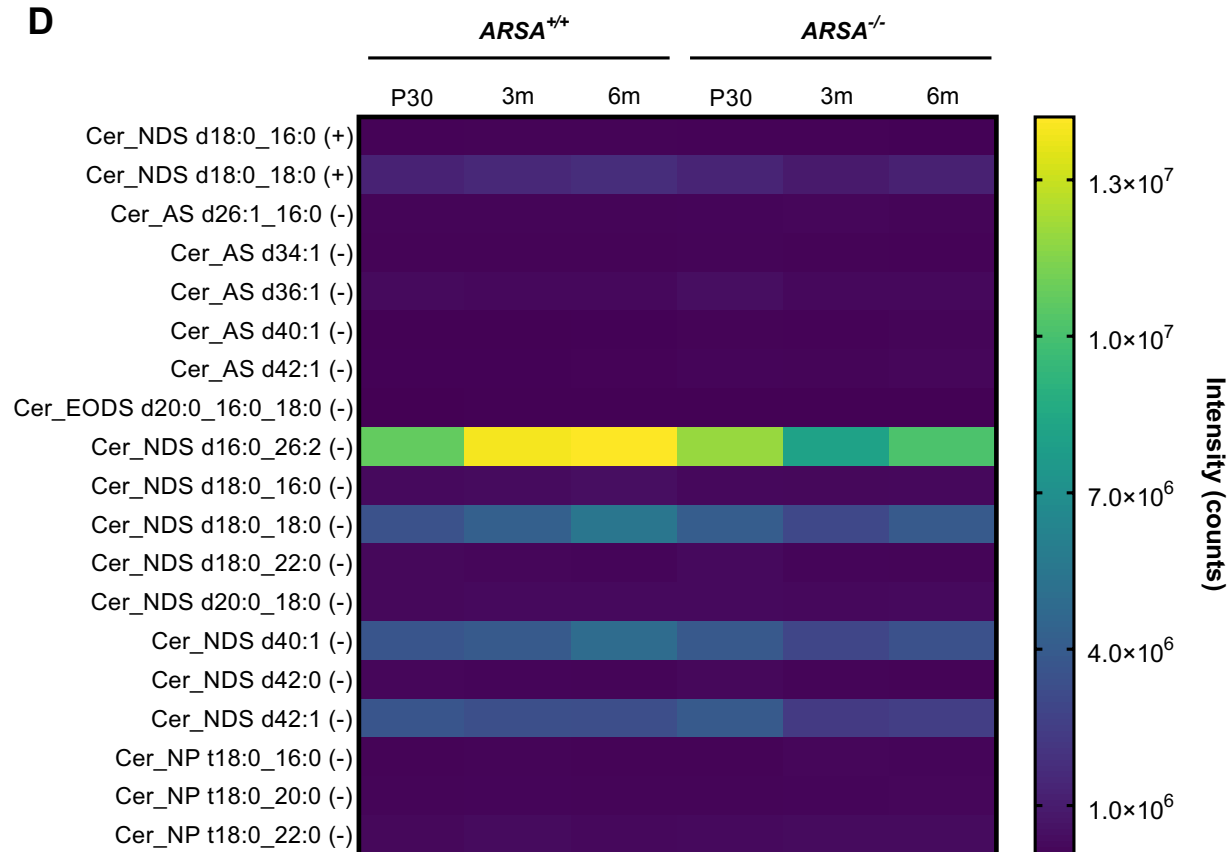

# E

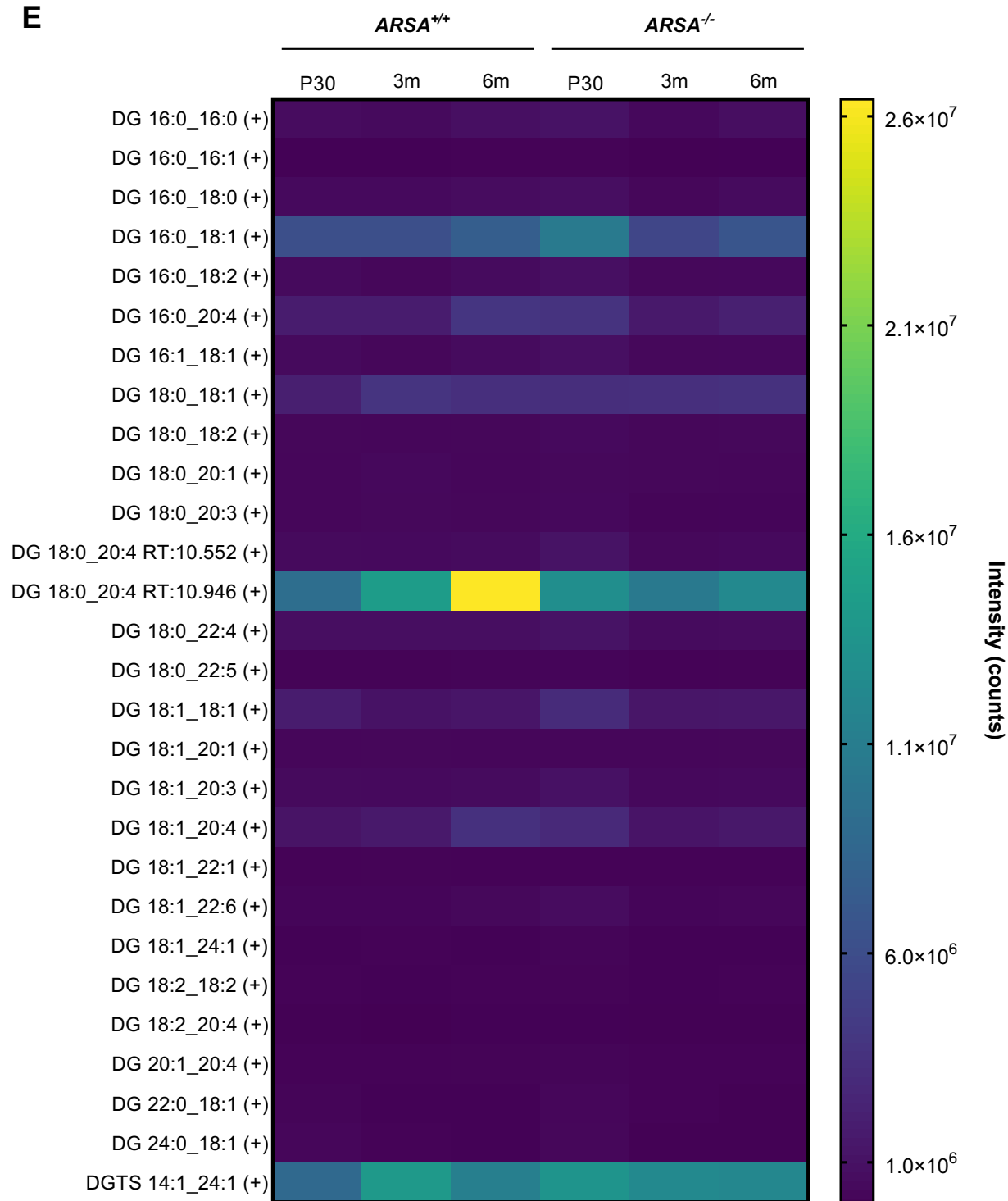

**F**

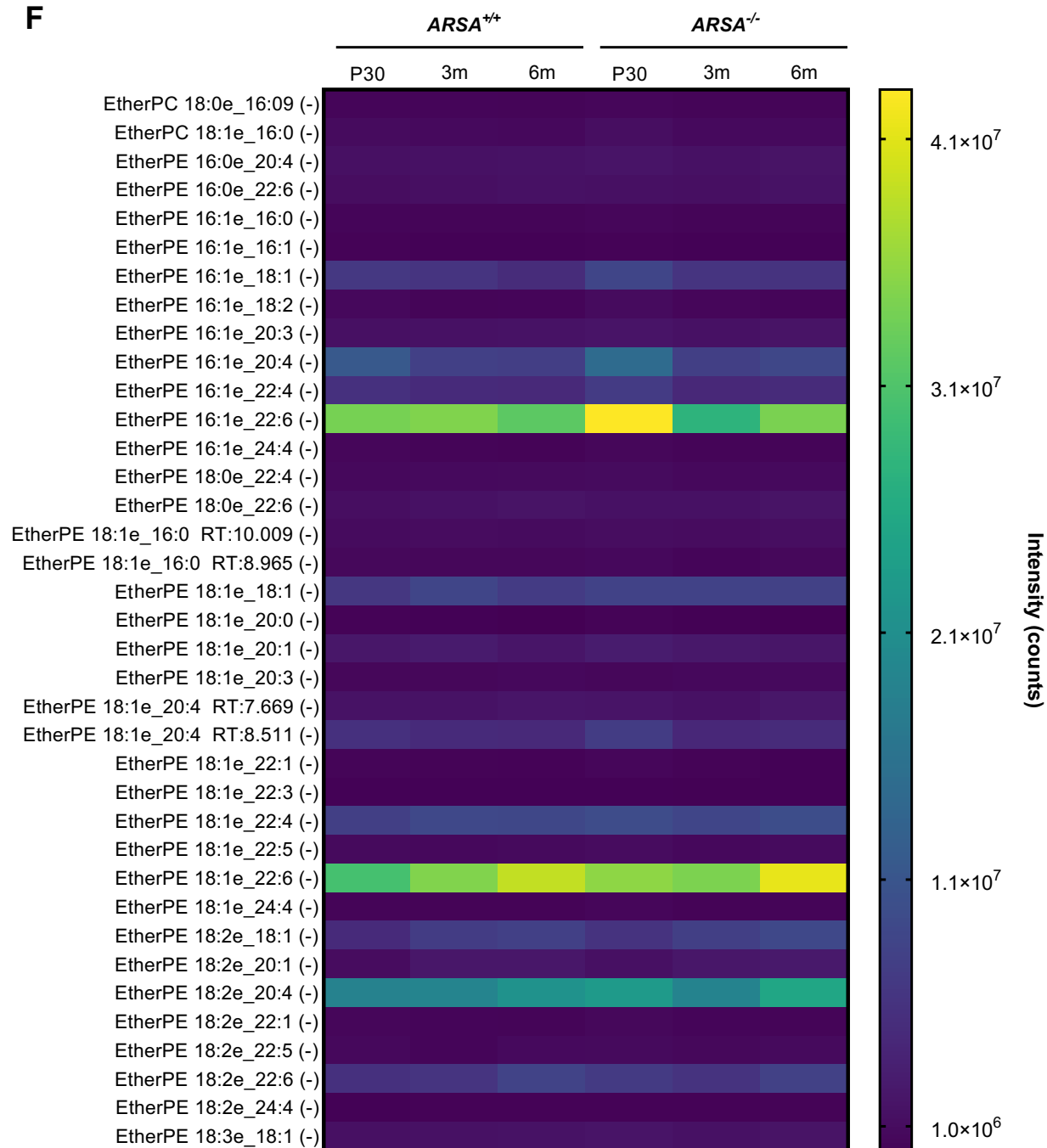

**G**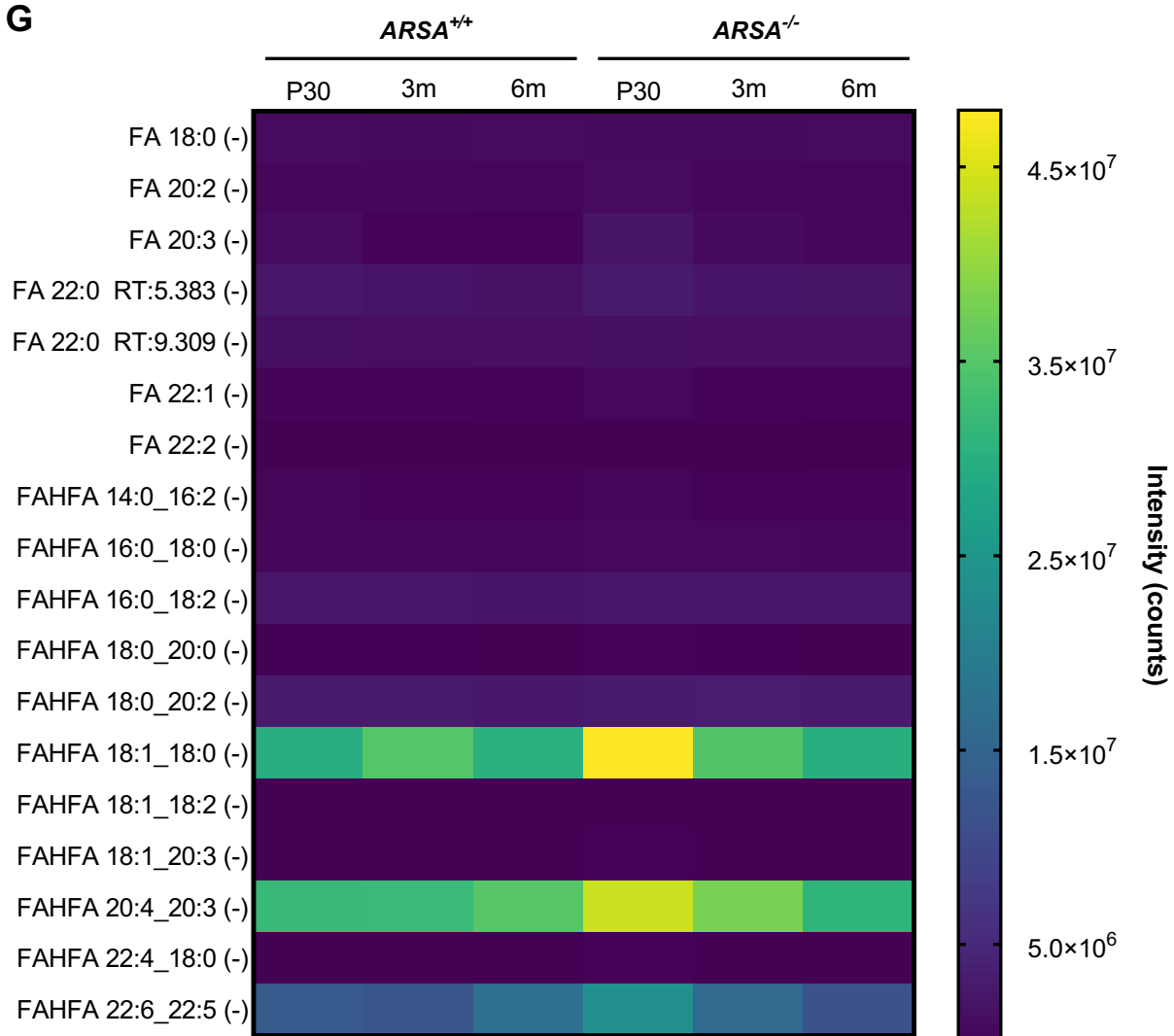

H

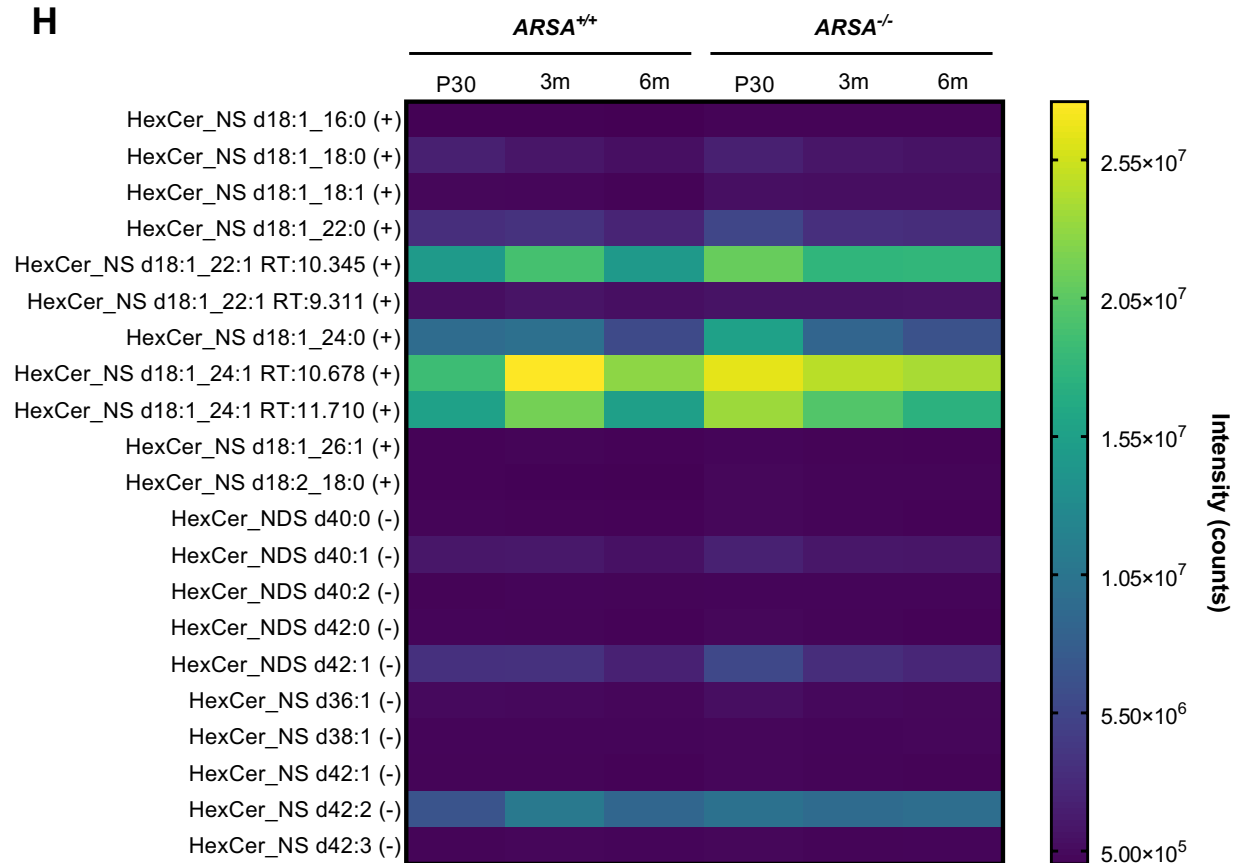

1

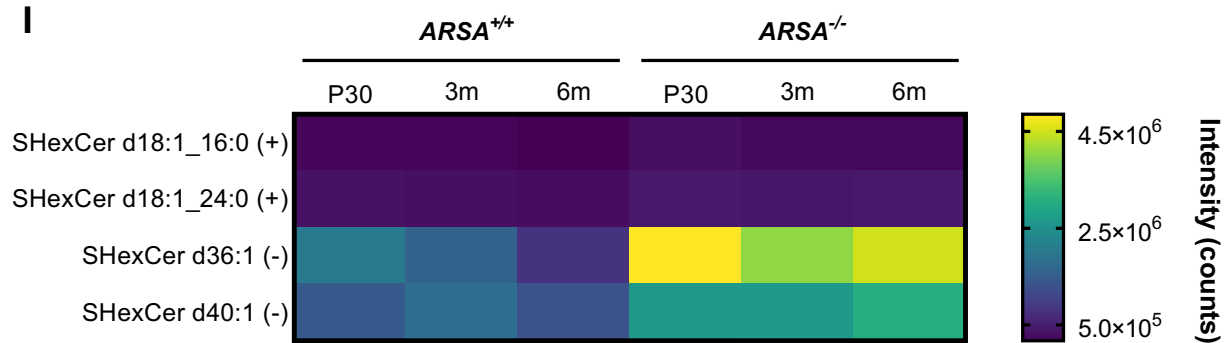

J

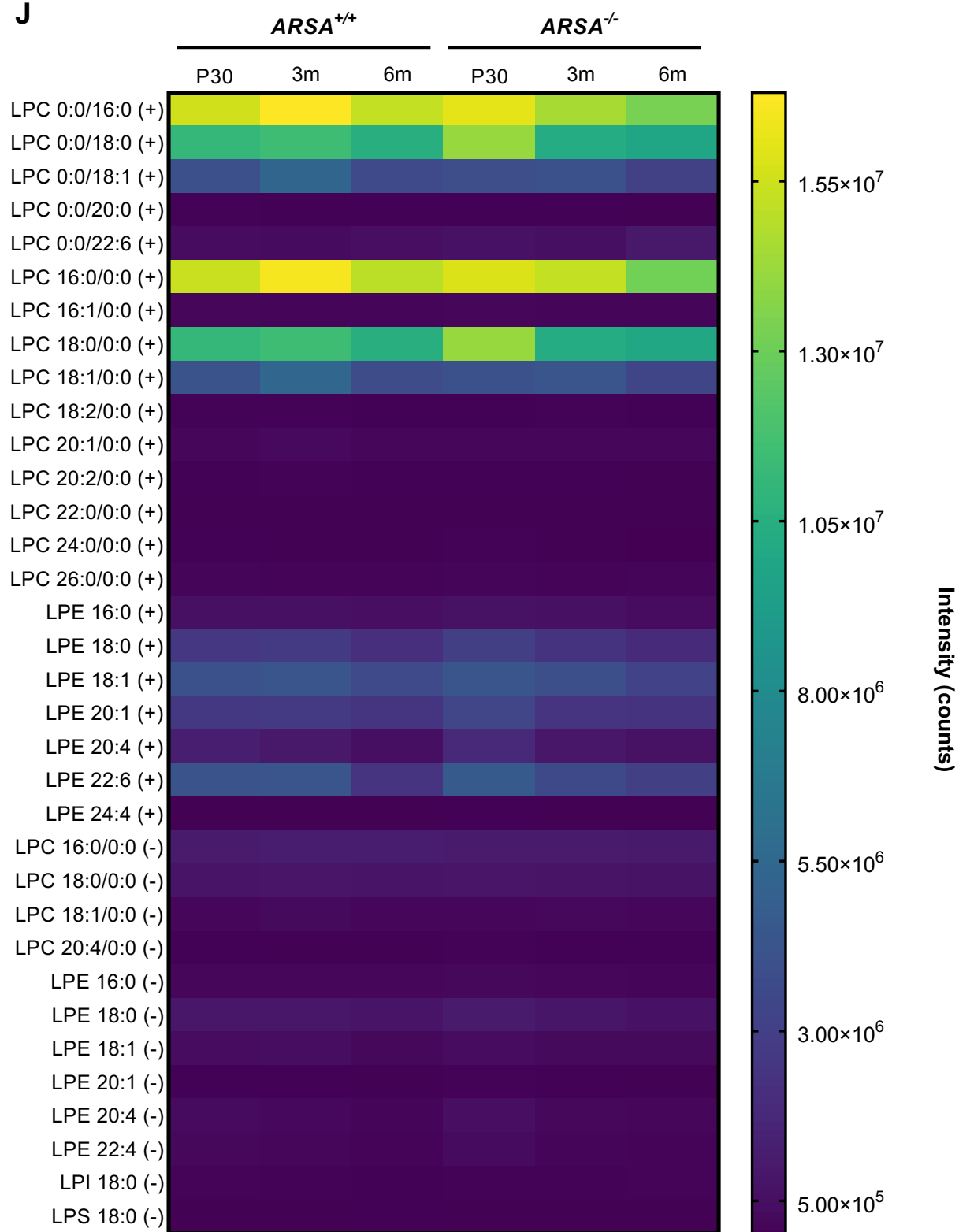

**K**

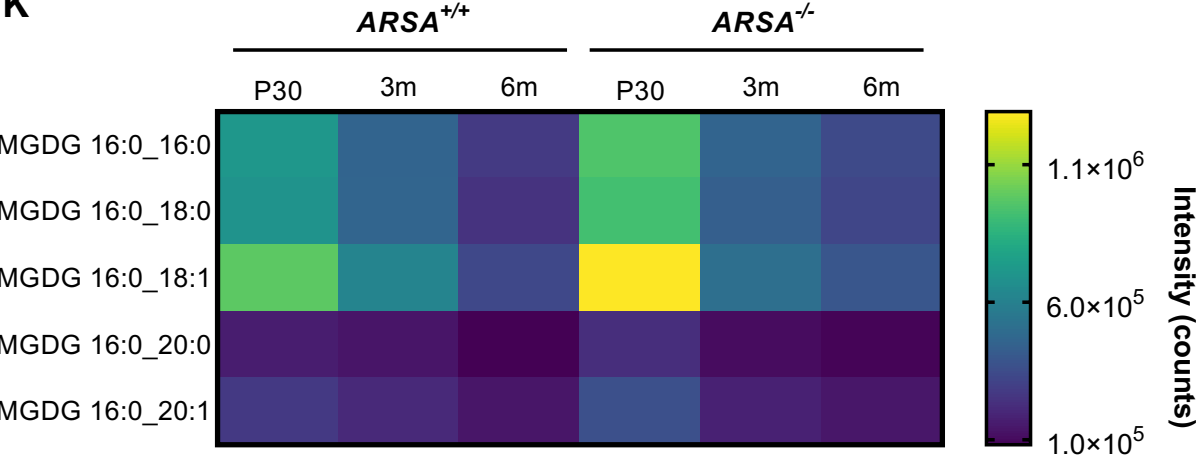

L

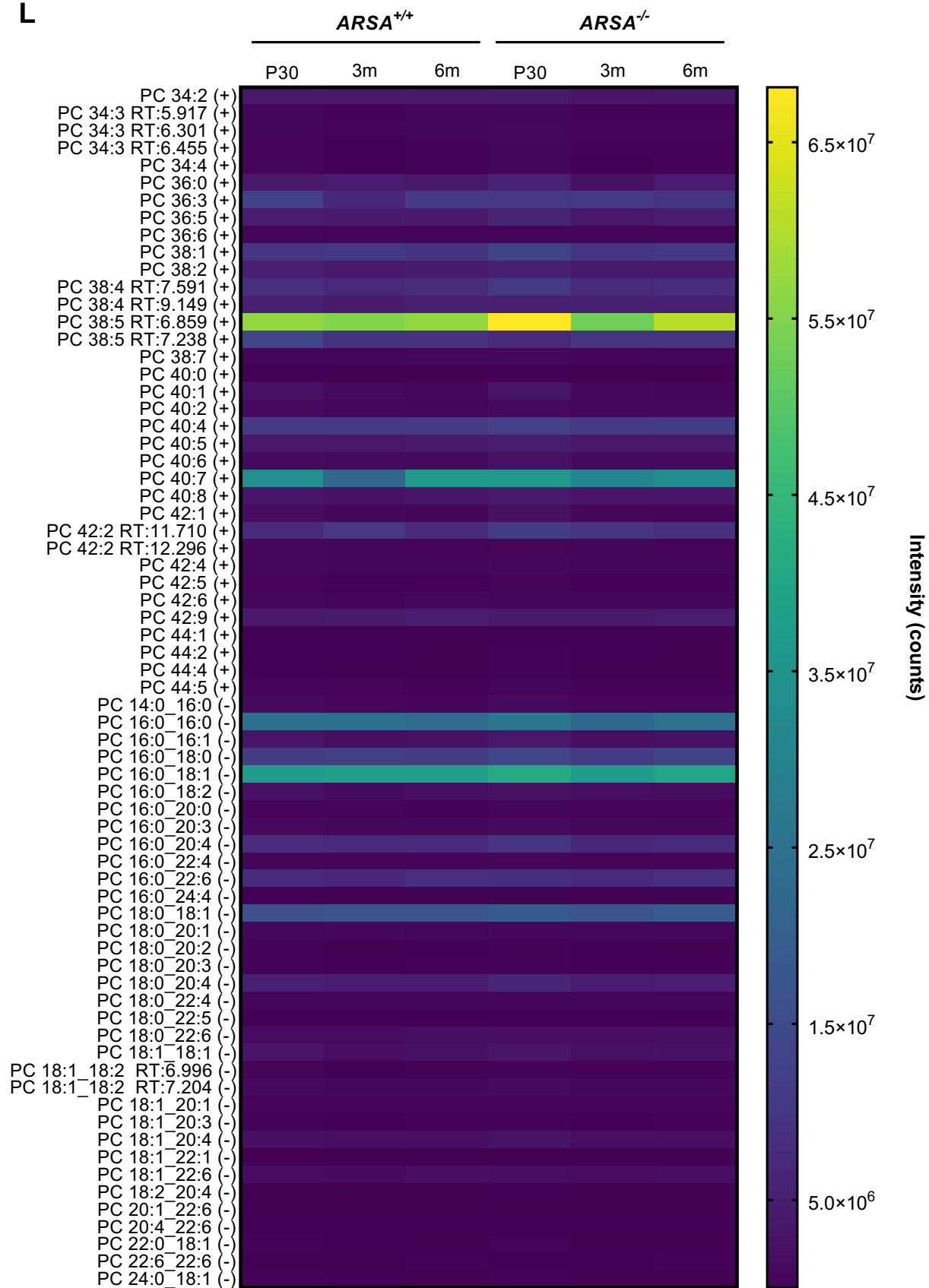

**M**

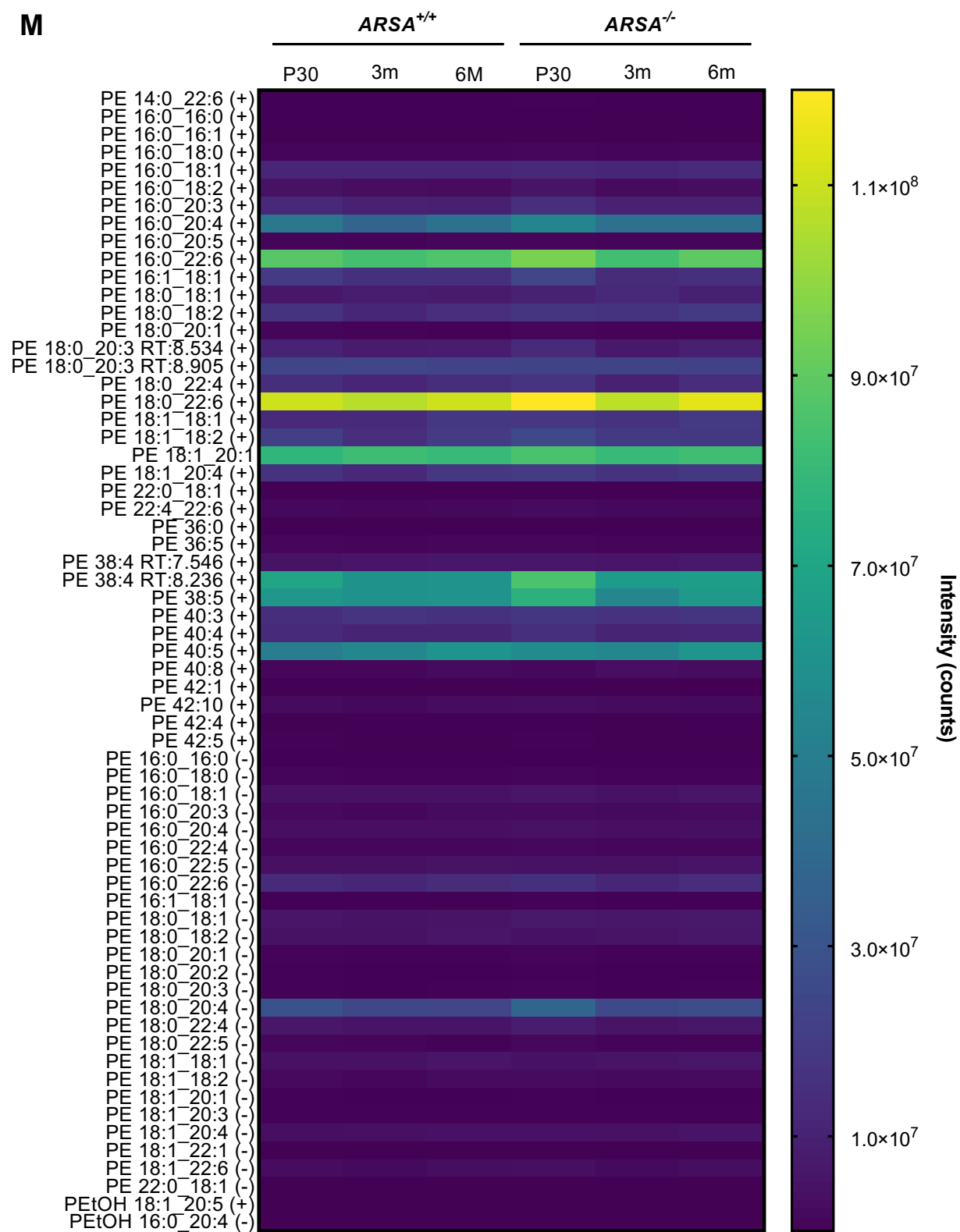

**N**

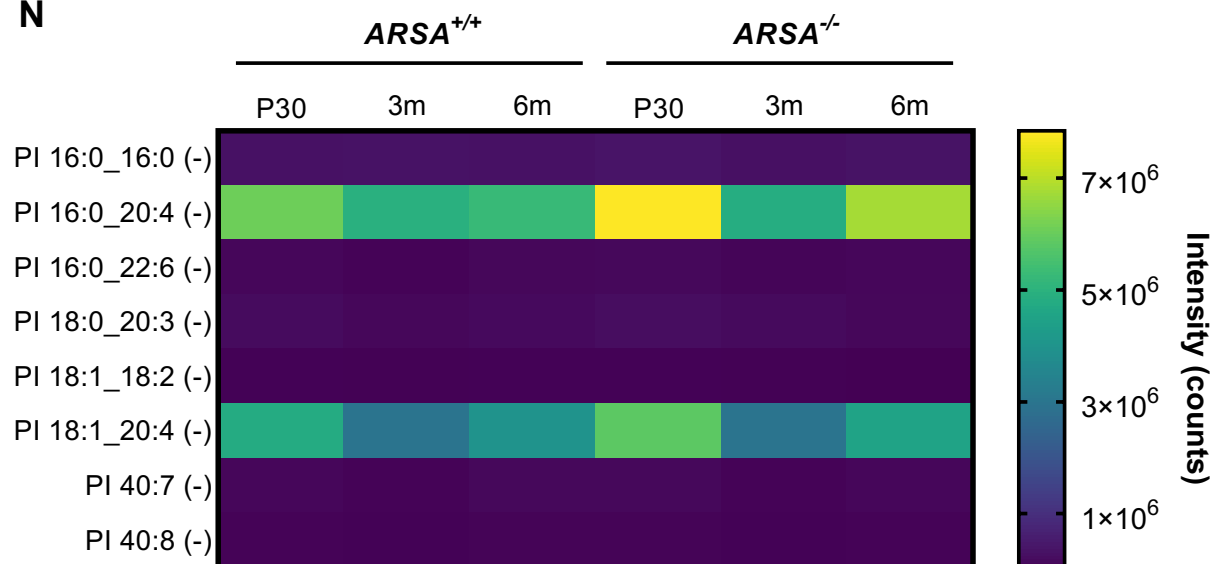

O

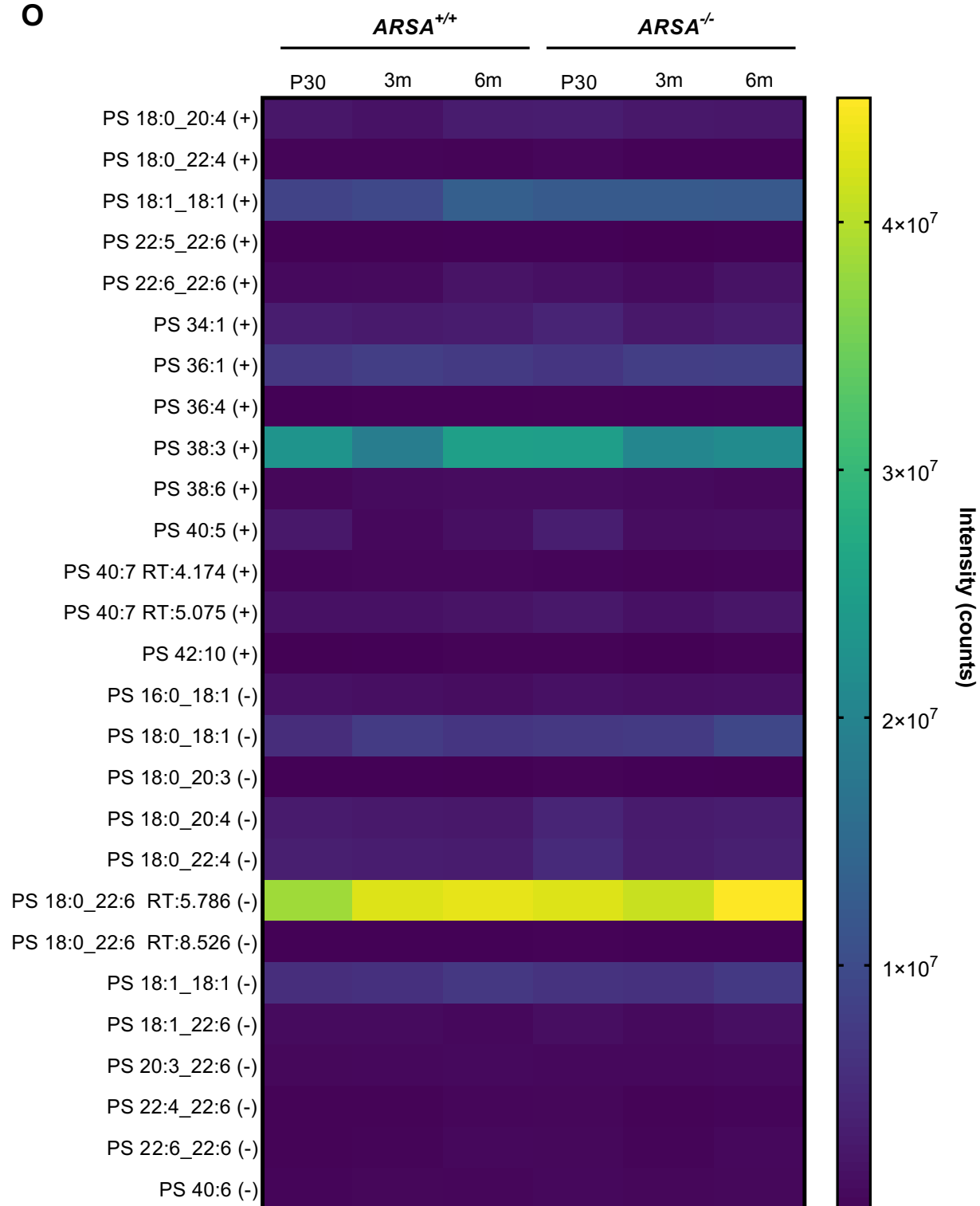

**P**

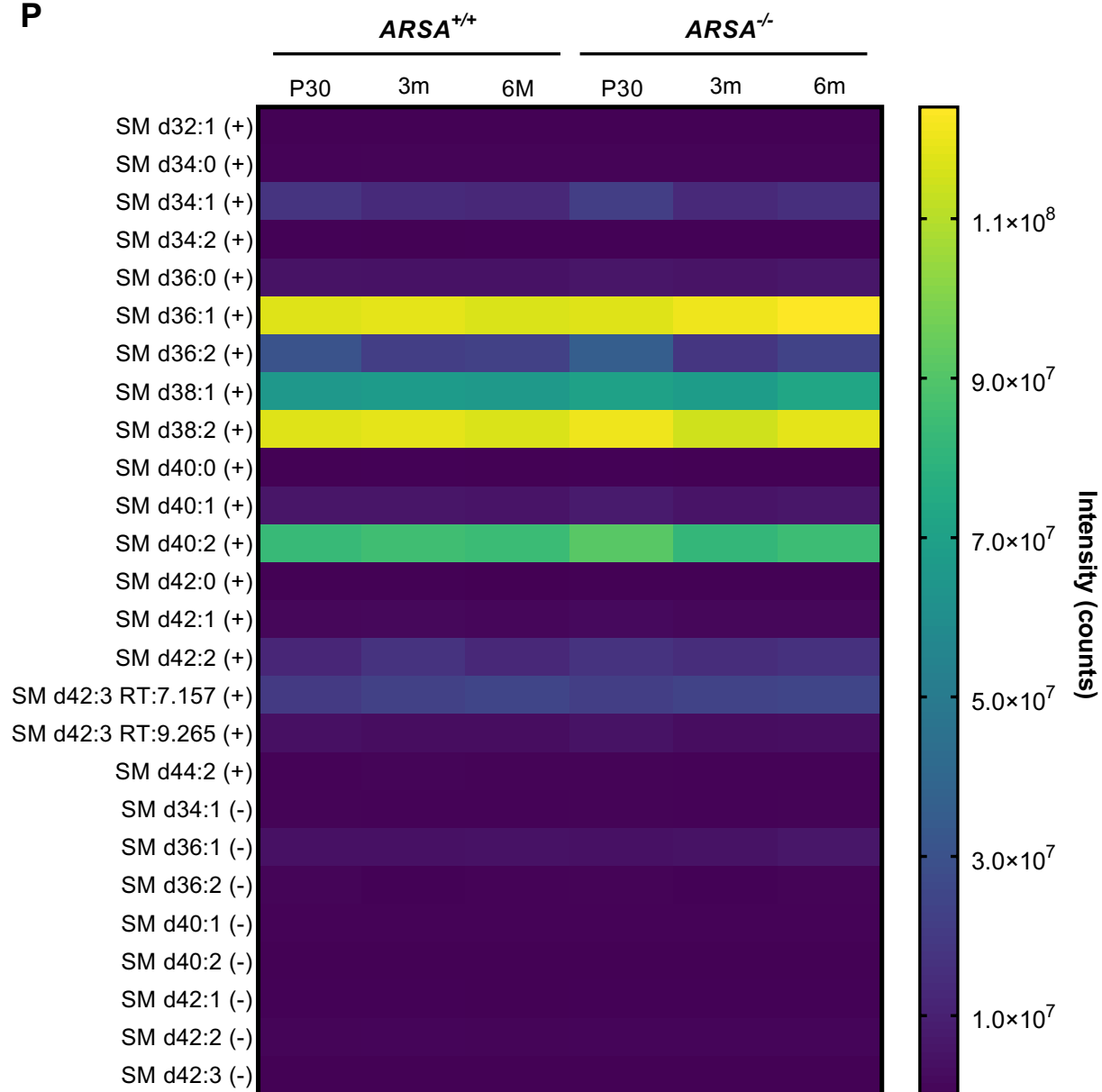

Q

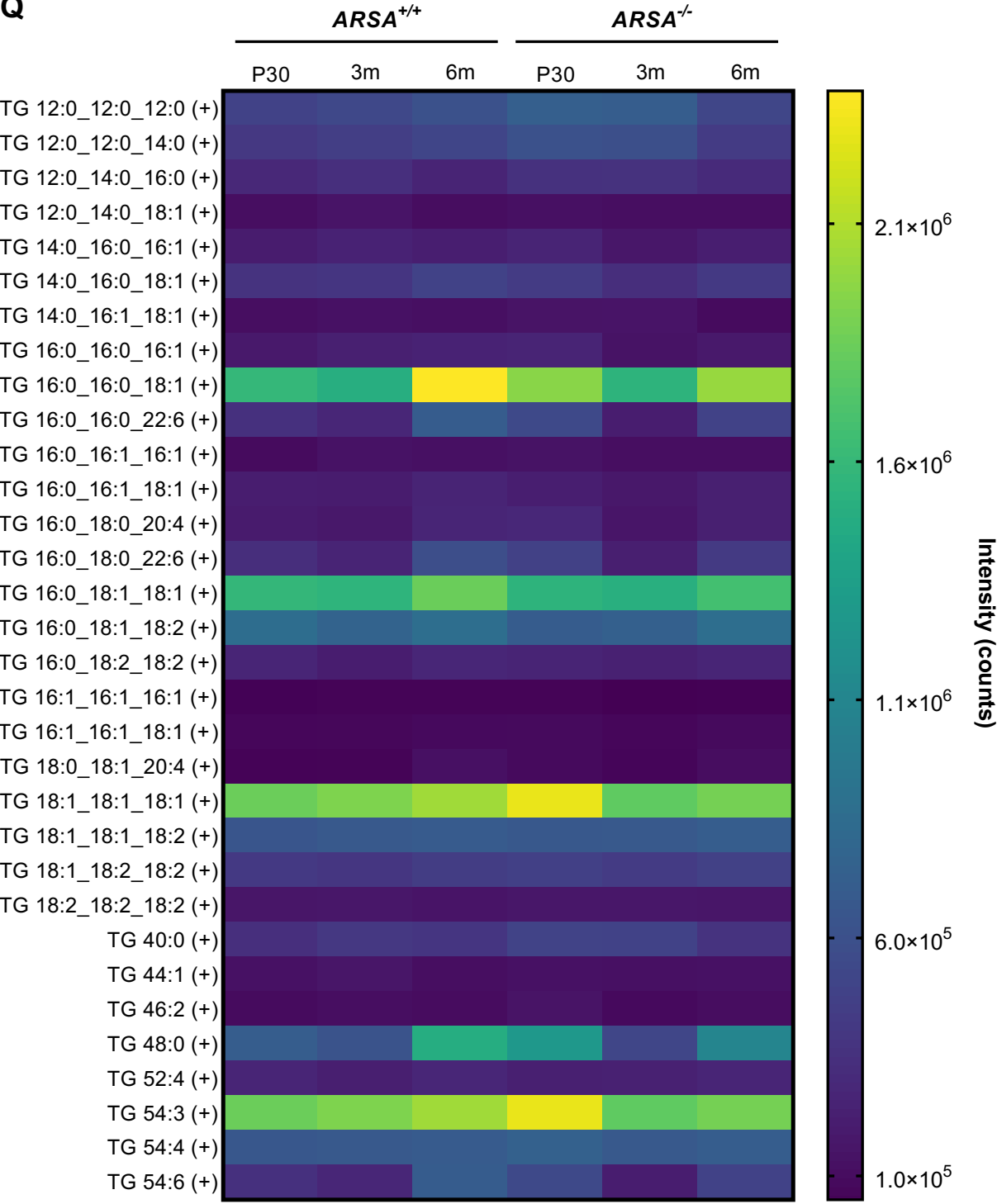

R

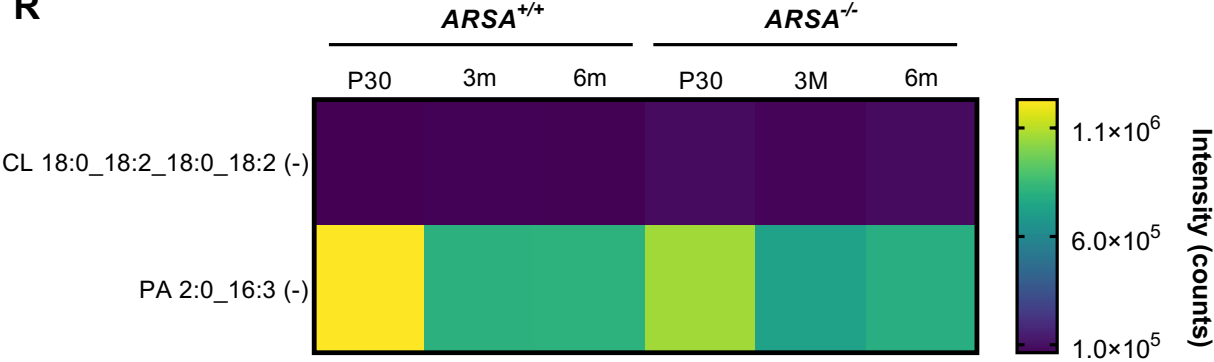

Supplement: Supplementary file 9 — Additional file 9. Heatmap representation of the intensity for lipids measured in brain derived EVs from MLD and control mice. Lipid species identified from the liquid chromatography mass spectrometry analysis of extracellular vesicles (EVs) for (A) acylcarnatine (ACar), (B) bis (monoacylglycero) phosphate (BMP)/ phosphatidylglycerol (PG), (C) ceramide non-hydroxyfatty acid-sphingosine (Cer_NS), (D) ceramide non-hydroxyfatty acid-dihydrosphingosine (Cer_NDS), (E) ceramide alpha-hydroxy fatty acid-sphingosine (Cer_AS), (F) ceramide esterified omega-hydroxy fatty acid-dihydrosphingosine (Cer_EODS), (G) (DG) diacylglycerol, (F) ether-linked phophatidylethanolamine (EtherPE), (G) fatty acid (FA), (H) fatty acid ester of hydroxyl fatty acid (FAHFA), (I) hexosylceramide non-hydroxyfatty acid-sphingosine (HexCer_NS), (J) hexosylceramide non-hydroxyfatty acid-dihydrosphingosine (HexCer_NDS), (K) Sulfatide (SHexCer), (L) lysophophatidylcholine (LPC), (M) lysophosphatidylethanolamine (LPE), (N) lysophosphatidylinositol (LPI), (O) lysophosphatidylserine (LPS), (P) monogalactosyldiacylglycerol (MGDG), (Q) phophatidylcholine (PC), (R) phatidylethanolamine (PE), (S) phosphatidylethanol (PEtOH), (T) phatidylinositol (PI), (U) phosphatidylserine (PS), (V) sphingomyelin (SM), (W) triacylglycerol (TG), (X) phosphatidic acid (PA) and (Y) cardiolipin (CL) lipid classes are shown for each biological replicate for control (ARSA+/+) and MLD (ARSA−/−) mice. Lipids noted with (+) or (−) were identified by fragmentation matching in positive or negative ion modes, respectively. [file 12944_2022_1644_MOESM9_ESM.pdf]

**A**

## Positive Ion Mode

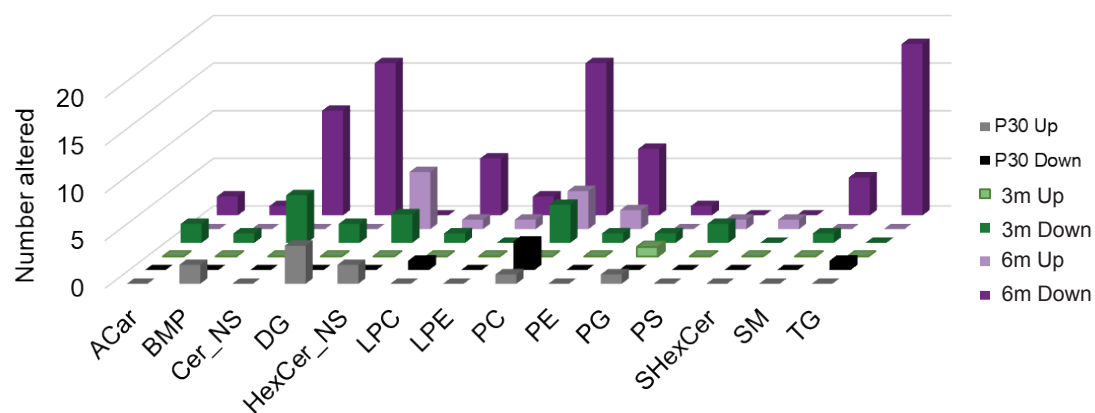

**B**

## Negative Ion Mode

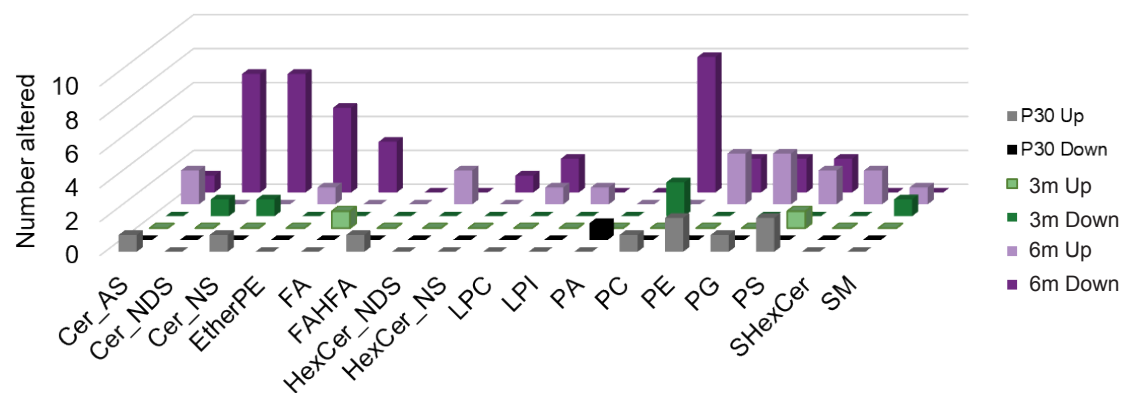

Supplement: Supplementary file 10 — Additional file 10. Plots showing the number of altered lipids per class measured in brain derived lipids enriched from MLD and control mice. Bar charts showing the number of lipid species significantly (p < 0.05) altered lipid species identified in extracellular vesicle (EV) lipid extracts measured via mass spectrometry analysis in (A) positive and (B) negative ion modes for acylcarnatine (ACar), bis (monoacylglycero) phosphate (BMP)/ phosphatidylglycerol (PG), ceramide non-hydroxyfatty acid-sphingosine (Cer_NS), ceramide non-hydroxyfatty acid-dihydrosphingosine (Cer_NDS), ceramide alpha-hydroxy fatty acid-sphingosine (Cer_AS), diacylglycerol (DG), ether-linked phophatidylethanolamine (EtherPE), fatty acid (FA), fatty acid ester of hydroxyl fatty acid (FAHFA), hexosylceramide non-hydroxyfatty acid-sphingosine (HexCer_NS), hexosylceramide non-hydroxyfatty acid-dihydrosphingosine (HexCer_NDS), sulfatide (SHexCer), lysophophatidylcholine (LPC), lysophosphatidylethanolamine (LPE), lysophosphatidylinositol (LPI), lysophosphatidylserine (LPS), monogalactosyldiacylglycerol (MGDG), phophatidylcholine (PC), phosphatidylethanolamine (PE), phosphatidylinositol (PI), phosphatidylserine (PS), sphingomyelin (SM) and triacylglycerol (TG) lipid classes at postnatal day 30 (P30), 3 months (3 m) and 6 months (6 m) of age for control (ARSA+/+) and MLD (ARSA−/−) mice. [file 12944_2022_1644_MOESM10_ESM.pdf]

A

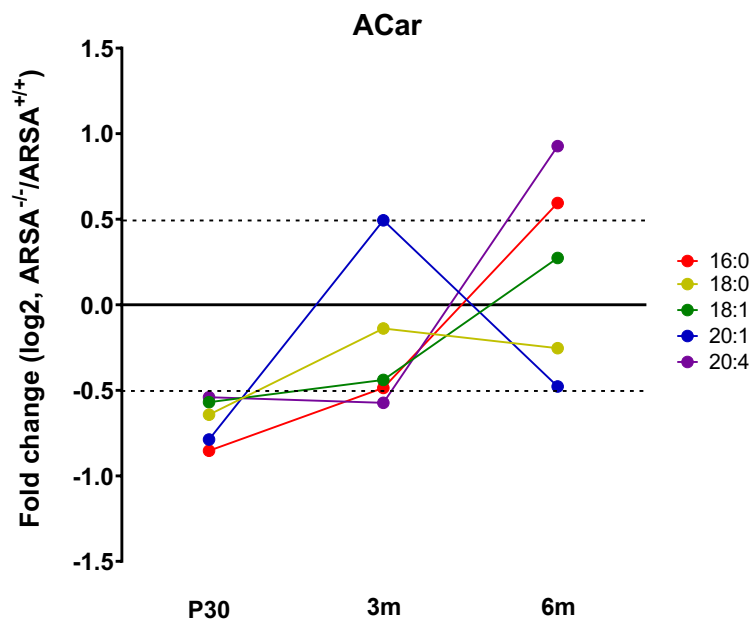

B

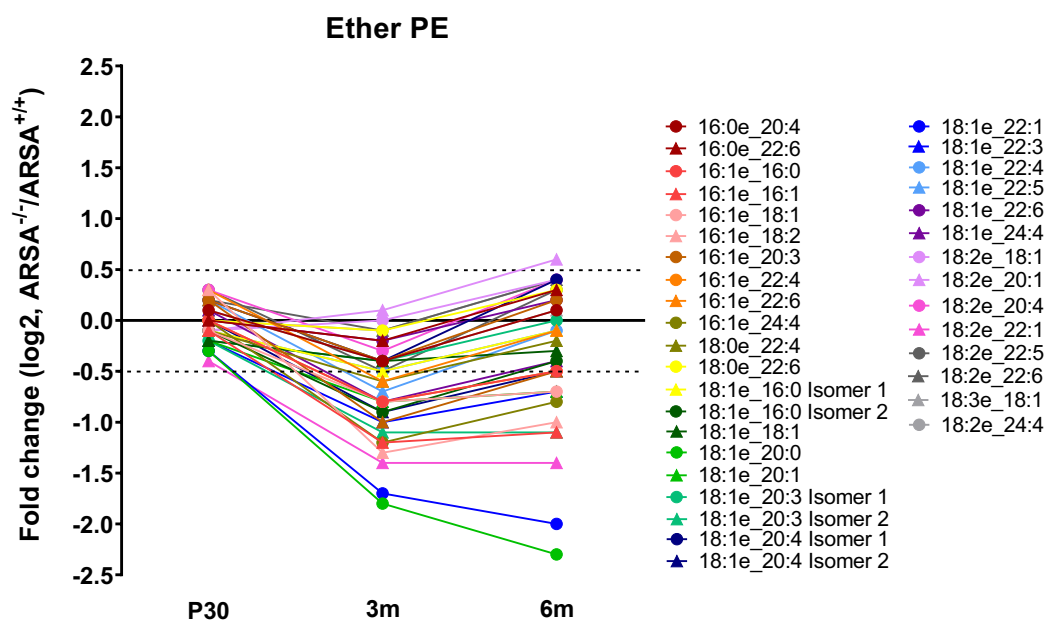

C

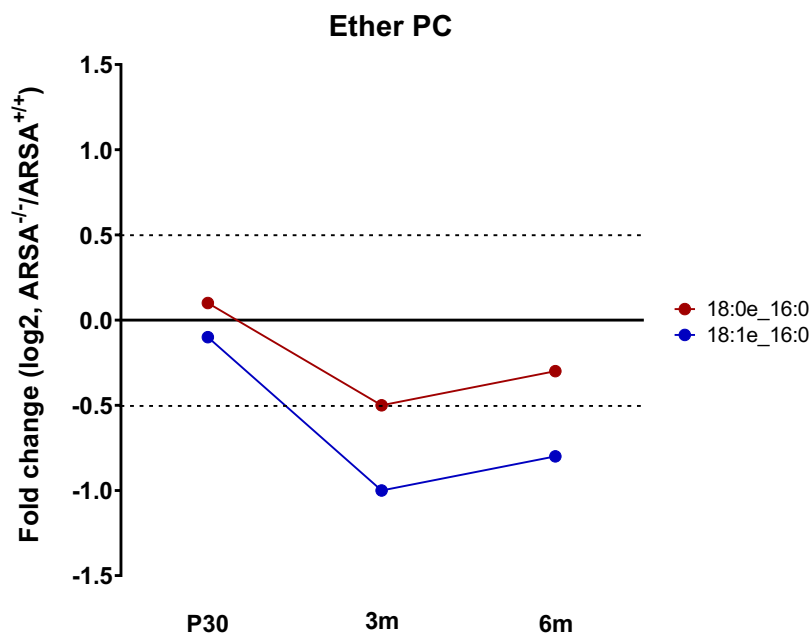

D

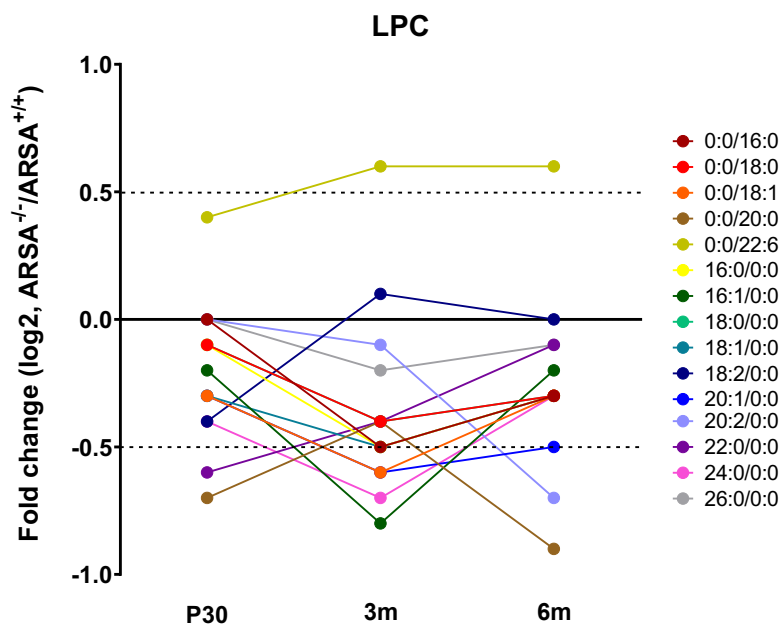

E

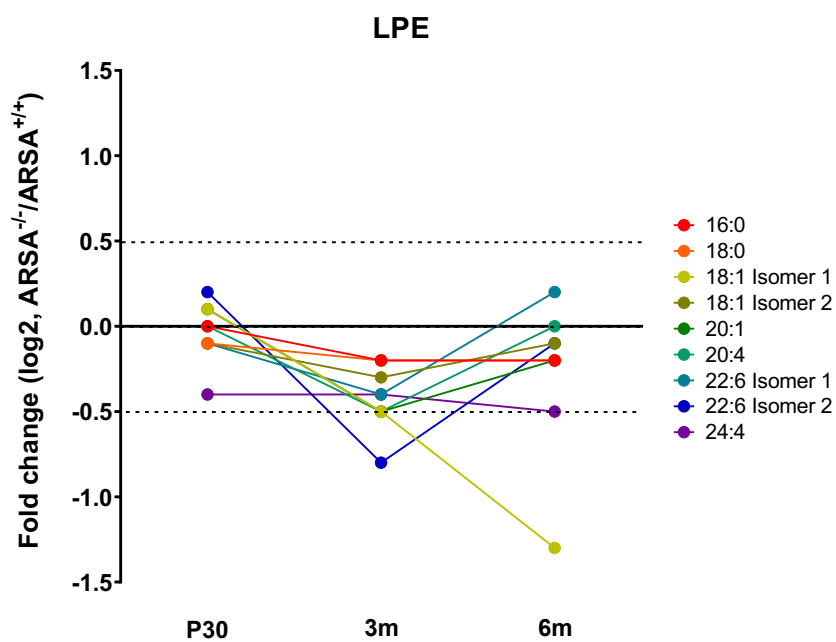

F

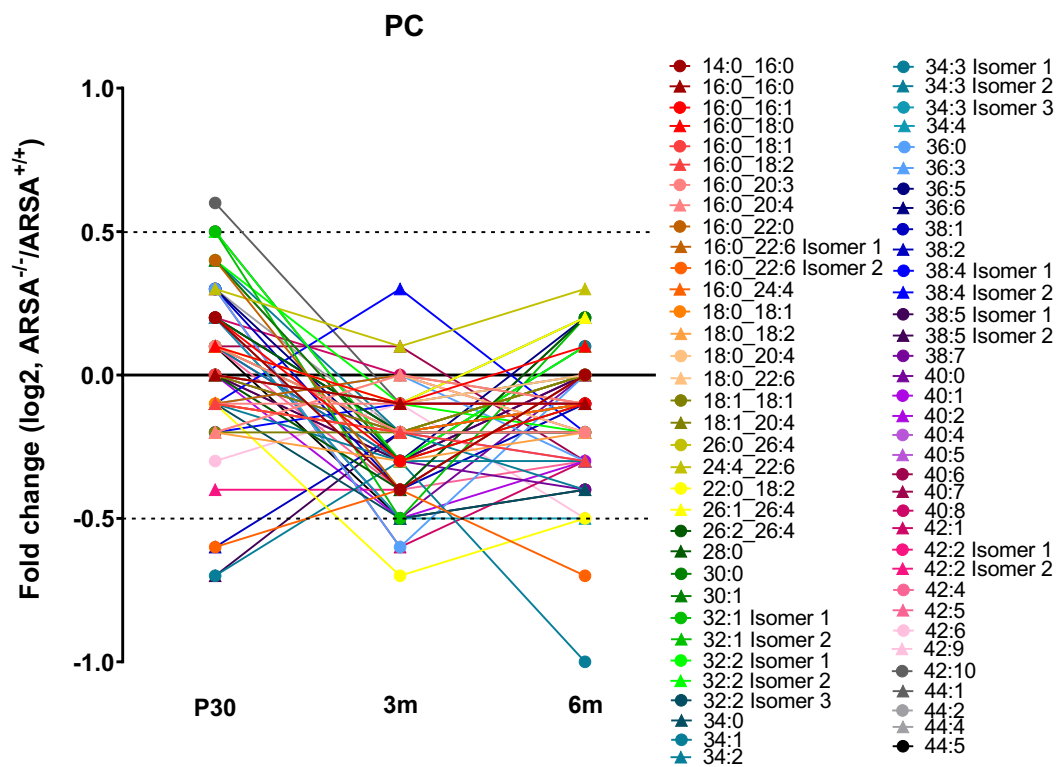

G

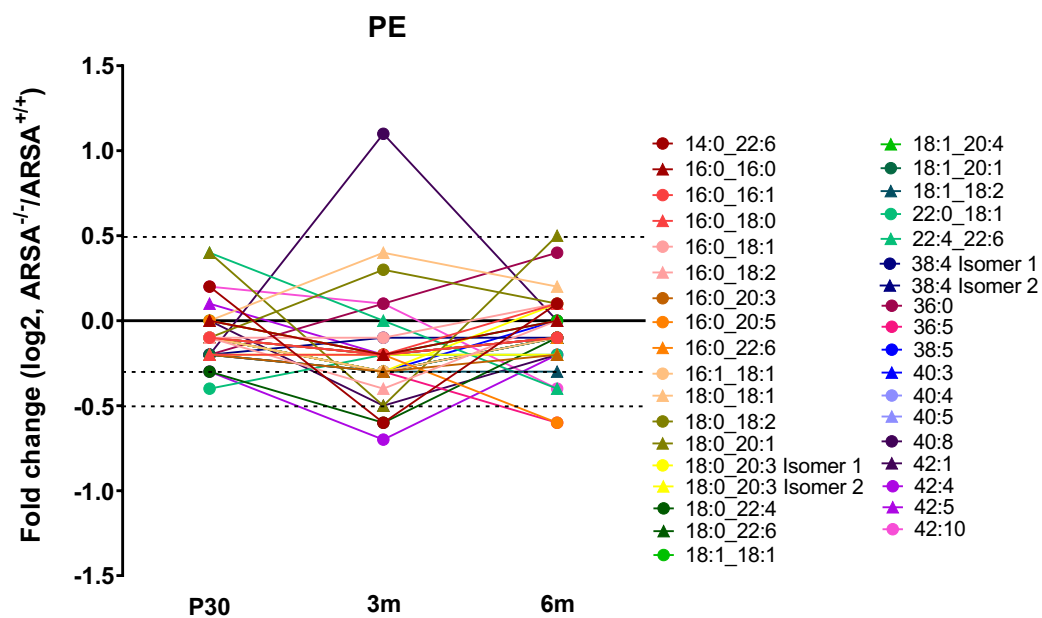

H

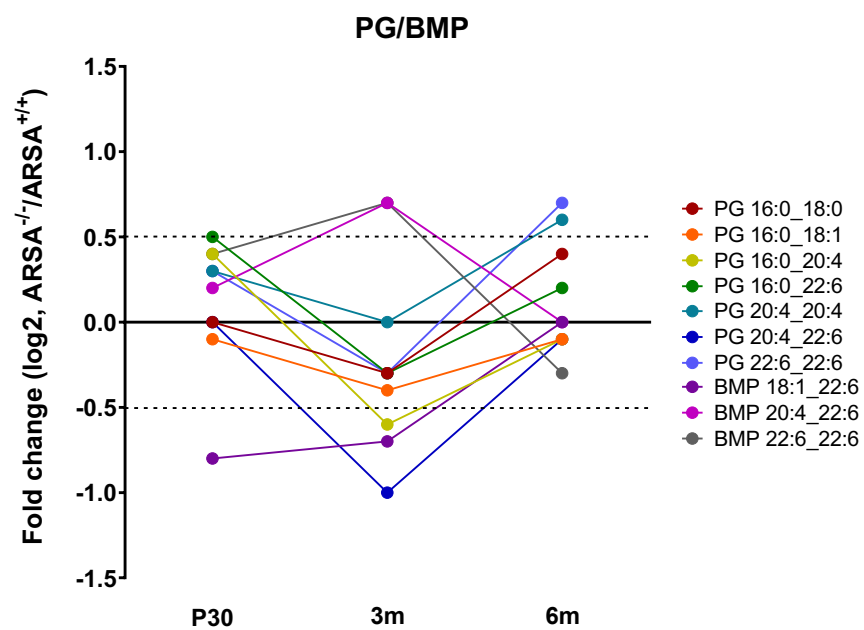

I

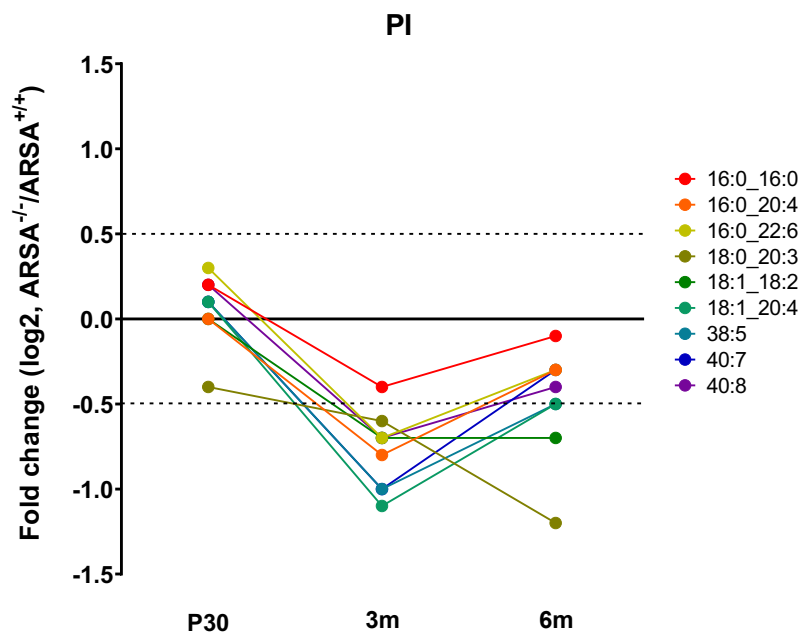

J

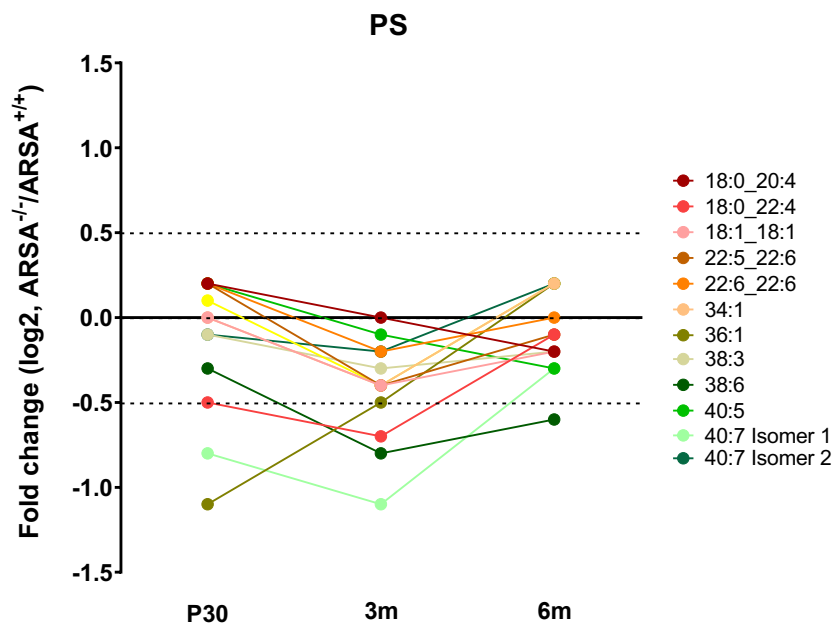

K

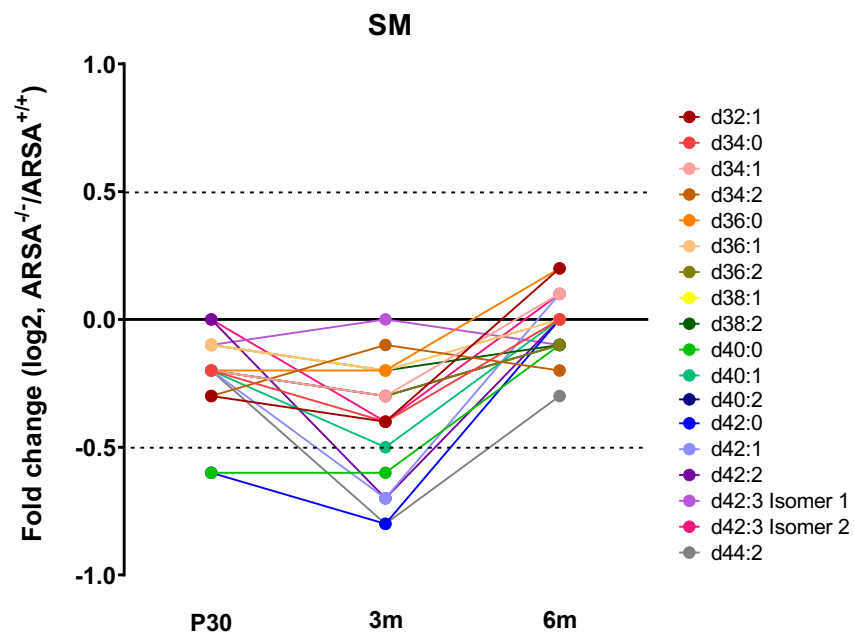

Supplement: Supplementary file 11 — Additional file 11. Derived lipid class that don’t show a correlation across time points of disease in MLD mice. Scatter plots for lipid classes that do not show a correlation across time points of disease in MLD mice. Fold change plotted for MLD (ARSA−/−) relative to control (ARSA+/+) mice at 30 postnatal days (P30), 3 months (3 m) and 6 months (6 m) of age for (A) acylcarnitine (ACar), (B) ether-linked phophatidylethanolamine (EtherPE), (C) ether-linked phophatidylphophatidylcholine (EtherPC), (D) lysophophatidylcholine (LPC), (E) lysophosphatidylethanolamine (LPE), (F) phophatidylcholine (PC), (G) phosphatidylethanolamine (PE), (H) phosphatidylinositol (PI), (I) phosphatidylglycerol (PG)/ bis (monoacylglycero) phosphate (BMP), (J) phosphatidylserine (PS) and (K) sphingomyelin (SM) lipid classes. [file 12944_2022_1644_MOESM11_ESM.pdf]
