# Supplementary material for: Lipidomic analysis identifies age-disease-related changes and potential new biomarkers in brain-derived extracellular vesicles from metachromatic leukodystrophy mice
Source: Lipids Health Dis. 2022 Mar 27;21:32. doi: 10.1186/s12944-022-01644-8 (PMC8962106; doi:10.1186/s12944-022-01644-8)
Supplement: Supplementary file 4 — Additional file 4. Summary of EVs size (mean and mode). [file 12944_2022_1644_MOESM4_ESM.pdf]

	**EV P30**	**EV P30**	**EV 3m**	**EV 3m**	**EV 6m**	**EV 6m**
	ASA+/+	***ASA-/-***	***ASA+/+***	***ASA-/-***	***ASA+/+***	***ASA-/-***
**Mean (nm)**	184.7<br>232.6<br>182.5	209.5<br>189.5<br>225.8	235.5<br>237.7<br>247.8	281.0<br>292.9<br>287.0	186.6<br>291.8<br>241	200.6<br>237.4<br>236.9
**Mode (nm)**	140.1<br>142.7<br>114.9	134.7<br>149.0<br>132.7	147.4<br>146.9<br>156.1	185.7<br>264.8<br>186.6	138<br>181.9<br>170	141.7<br>158.7<br>158.1
